# Genomic, Transcriptomic and Enzymatic Insight into Lignocellulolytic System of a Plant Pathogen *Dickeya* sp. WS52 to Digest Sweet Pepper and Tomato Stalk

**DOI:** 10.3390/biom9120753

**Published:** 2019-11-20

**Authors:** Ying-Jie Yang, Wei Lin, Raghvendra Pratap Singh, Qian Xu, Zhihou Chen, Yuan Yuan, Ping Zou, Yiqiang Li, Chengsheng Zhang

**Affiliations:** 1 Marine Agriculture Research Center, Tobacco Research Institute of Chinese Academy of Agricultural Sciences, Qingdao 266101, China; yingjieyoung@sina.com (Y.-J.Y.); yuanyuan03@caas.cn (Y.Y.); zouping@caas.cn (P.Z.); liyiqiang@caas.cn (Y.L.); 2 Tobacco Research Institute of Nanping, Nanping, Fujian 353000, China; lw160718@163.com (W.L.); xq711126@163.com (Q.X.); hhh0599@163.com (Z.C.); 3 Department of Research & Development, Biotechnology, Uttaranchal University, Dehradun 248007, India

**Keywords:** *Dickeya* sp., lignocellulose degradation, CAZy, pectin degradation, transcriptome sequencing, monosaccharide analysis

## Abstract

*Dickeya* sp., a plant pathogen, causing soft rot with strong pectin degradation capacity was taken for the comprehensive analysis of its corresponding biomass degradative system, which has not been analyzed yet. Whole genome sequence analysis of the isolated soft-rotten plant pathogen *Dickeya* sp. WS52, revealed various coding genes which involved in vegetable stalk degradation-related properties. A total of 122 genes were found to be encoded for putative carbohydrate-active enzymes (CAZy) in *Dickeya* sp. WS52. The number of pectin degradation-related genes, was higher than that of cellulolytic bacteria as well as other *Dickeya* spp. strains. The CAZy in *Dickeya* sp.WS52 contains a complete repertoire of enzymes required for hemicellulose degradation, especially pectinases. In addition, WS52 strain possessed plenty of genes encoding potential ligninolytic relevant enzymes, such as multicopper oxidase, catalase/hydroperoxidase, glutathione S-transferase, and quinone oxidoreductase. Transcriptome analysis revealed that parts of genes encoding lignocellulolytic enzymes were significantly upregulated in the presence of minimal salt medium with vegetable stalks. However, most of the genes were related to lignocellulolytic enzymes, especially pectate lyases and were downregulated due to the slow growth and downregulated secretion systems. The assay of lignocellulolytic enzymes including CMCase and pectinase activities were identified to be more active in vegetable stalk relative to MSM + glucose. However, compared with nutrient LB medium, it needed sufficient nutrient to promote growth and to improve the secretion system. Further identification of enzyme activities of *Dickeya* sp.WS52 by HPLC confirmed that monosaccharides were produced during degradation of tomato stalk. This identified degradative system is valuable for the application in the lignocellulosic bioenergy industry and animal production.

## 1. Introduction

*Solanaceae* vegetable crops, especially sweet pepper and tomato, produces significant amount of crop residues, which is not directly used as feed, due to their low digestibility [1,2]. Therefore, most of them are left to decay in the fields or are burnt [3]. Treatment of agricultural wastes is not only a concern for the environment but it also affects the development of sustainable agriculture [4,5,6]. Exploring microorganisms and enzymes associated with the degradation of biomass by traditional methods or functional metagenomic approaches can have great potentials for sustainable agriculture and biofuel production [4,6,7,8]. These vegetable wastes are made-up of cellulose, hemicellulose and lignin. Pectin- a hemicellulose, a plant cell wall, polysaccharide with structural backbones and is rich in galacturonic acid residues. It contributes 2–35% of plant dry mass [9,10]. The most common form of pectin is homogalacturonan [11], A chain of alpha-1,4 linked galacturonic acid residues. The galacturonic acid residues are generally methylesterified or acetylated [9,12]. Other forms of pectin include rhamnogalacturonan and xylogalacturonan [13]. Rhamnogalacturonan I, the second most common form of pectin, contains a backbone of repeating dimers of α-1,6 linked galacturonic acid and α-1,4 linked rhamnose sugars. Rhamnogalacturonans have highly varied branched structures usually featuring side chains of α-1,5-linked arabinan or α-1,4-linked galactan connected via the rhamnose residues [9,14].

Pectinase is a collection of enzymes that catalyze reactions at a variety of sites on a pectin molecule [15]. Pectin esterase includes pectin methylesterase and pectin acetylesterase that catalyzes the de-esterification of galacturonic acid residues releasing free methanol or acetic acid [16,17]. Pectin esterase is the first step enzyme to digest pectin and to produce pectate and/or polygalacturonate. It can increase the accessibility of pectin to polygalacturonase or pectate lyase [18,19]. Pectate lyases cleave the α1,4-glycosidic linkages of polygalacturonate, generating oligogalacturonides with C4-C5 unsaturation at the nonreducing end. Pectate lyases are usually specific for the nonmethylated polysaccharide or for pectins with a low degree of methyl esterification [20]. Pectate lyases are classified into different families of polysaccharide lyases (PL) according to their primary amino acid sequences [21,22,23], listed on the homepage http://www.cazy.org/. Polygalacturonase is responsible for the hydrolytic breakdown of the polygalacturonic acid backbone of pectin to produce galacturonic acid oligomers or monomers [24]. Pectinase is used industrially to process fibers for textiles, to clarify wine and fruit juices in pulp and paper processing and as supplement in animal feed [1,25,26,27,28]. While a wide range of plants produce pectinase [29] commercial production is usually performed by microbial fermentation [15,30,31,32,33,34]. Till date pectinases have been isolated from various microbial sources such as bacteria [35], yeast [36], fungi [37] and actinomycetes [38] for broad application.

*Dickeya* causes soft-rot disease in a wide range of plant hosts, including many economically important vegetables such as potato and tomato [39]. Soft rot is due to the secretion of a battery of pectinases which acts by destroying the integrity of the plant cell wall [32]. As pectin acts as “glue” to stabilize the cell wall, it’s rapid degradation leads to cell lysis and release of the intracellular components [40]. Cellulase, xylanase, lignin-related enzymes and additional plant cell wall degrading enzymes are also simultaneously secreted by the bacteria [41] and can complement the pectinase activity regarding the breakdown of the cell wall components. Most of the pectinases and cellulases are secreted into the external medium via a common secretion system, The type II OutK secretion systems [42]. Among all these degrading enzymes, pectate lyases have a predominant role in plant tissue maceration. Until now, there are lots of *Dickeya* species isolated and identified. As a plant pathogen, *Dickeya* sp. has complex regulatory pathways which allow fine-tuning of gene expression to modulate its pectinolytic machinery [41,43,44,45]. There are some reports to compare the transcription pattern in free-living and plant tissues infection phase [43,46,47]. However, they are focused on the mechanism of plant pathogen none of strains or enzymes from *Dickeya* was analyzed by multi-omics techniques for applying it to industry. 

## 2. Materials and Methods

### 2.1. Raw Materials and Chemicals

After the harvest of vegetables, the stems along with the fresh leaves of sweet pepper and tomato were collected from the greenhouse in Qingzhou, Shandong Province, China, in January 2018. The biomass was oven dried, ground and sieved to obtain particles with 40 meshes. Standard D-glucose, D-galactose, D-mannose, D-xylose, L-rhamnose, L-fucose, D-glucuronic acid and galacturonic acid were purchased from Sigma- Aldrich Limited. All chemicals and reagents were of analytical grade.

### 2.2. Bacteria Strain and Culture Conditions

*Dickeya* sp. WS52 was isolated from soft-rot stem zone of tobacco growing in Nanping (27.63 N 118.04 E), Fujian, China. This strain has pectin degrading potentiality which caused the soft-rot disease (unpublished data). The culture was first grown in Luria Bertani (LB) and biomass was pelleted, washed with phosphate saline buffer and used to inoculate Minimal Salt Medium (MSM) for liquid fermentation with 1.0% or 5.0% tomato stalk or pepper stalk as sole carbon source (pH 7.0). MSM containing 0.5 g NaCl, 0.5 g MgSO_4_·7H_2_O, 0.1 g CaCl_2_·H_2_O, 3 g (NH_4_)_2_SO_4_, 1.1 g Na_2_HPO_4_, 0.25 g KH_2_PO_4_, 10 mg FeSO_4_·7H_2_O, 0.64 mg Na_2_EDTA·3H_2_O, 0.1 mg ZnCl_2_, 0.015 mg H_3_BO_3_, 0.175 mg CoCl_2_·6H_2_O, 0.15 mg Na_2_MoO_4_·2H_2_O, 0.02 mg MnCl_2_·4H_2_O, and 0.01 mg NiCl_2_·6H_2_O per liter (pH 7.0) was used [48]. MSM + glucose medium contains 1% glucose in MSM liquid medium. CTT medium contains casein peptone 1.0%, MgSO_4_·7H_2_O 8 mM, Tris-HCl (pH 7.6) 10 mM, KH_2_PO_4_/K_2_HPO_4_ (pH 7.6) 1 mM, the final pH was 7.6. The flasks were incubated on a rotary shaker having temperature 30 °C and rpm 180. All experiments were conducted in triplicate.

### 2.3. Bacterial Identification by 16S rRNA and Draft Genome Sequencing of Dickeya sp. WS52

The total gDNA of *Dickeya* sp. WS52 was extracted using a TIANamp Bacterial DNA kit (Tiangen Biotech Co. Ltd., Beijing, China) according to the manufacturer’s protocol. Draft genome sequencing was completed by Mega Genomics Co., Ltd. (Beijing, China) in an Illumina (San Diego, CA, USA) HiSeq 2500 platform. The resulting raw reads were then assembled using the SOAP de novo assembler version 2.04. Gene prediction was performed using the NCBI Prokaryotic Genome Annotation Pipeline server. The exist of signal peptide in protein sequence was identified by SignalP4.0 (http://www.cbs.dtu.dk/services/SignalP/) and Secretome P2.0 Server (http://www.cbs.dtu.dk/services/SecretomeP/) for some pectin degradation related proteins.

The 16S rRNA gene (approximately 1400 bp) was amplified by colony PCR using the universal primer pair 27F (5’-AGAGTTTGATCCTGGCTCAG-3’) and 1492R (5’-GGTTACCTTGTTACGACTT-3’). Single colonies of each selected isolate grown on LB medium were re-suspended in 50 μL of double-distilled water, boiled at 100 °C for 10 min. These solutions were used as templates. A PCR (50 µL total volume) was performed using KOD-plus-Neo DNA polymerase (Toyobo, Japan). The PCR products were purified and directly sequenced using the same primer pair (Tsingke Biotechnology Co. ltd, Qingdao, China) and the assembled sequence (1367 bp) was compared to the EzBioCloud database [49].

### 2.4. Transcriptome Sequencing

#### 2.4.1. Sample Collection and RNA Preparation

The bacterial cells were collected in two conditions: exponentially grown culture in LB medium and MSM medium supplemented with 1.0% tomato stalk powder and/or sweet pepper stalk powder. Three independent biological repeats were conducted for each treatment. Total RNA was extracted using TRIzol^®^ Reagent (Invitrogen, Carlsbad, CA, USA) according to the manufacturer’s protocol. All RNA samples were treated with DNase I (TaKara, Dalian, China). RNA purity was checked using the NanoPhotometer^®^ spectrophotometer (IMPLEN, Westlake Village, CA, USA). RNA concentration was measured using the Qubit^®^ RNA Assay Kit in a Qubit^®^ 2.0 Flurometer (Life Technologies, Camarillo, CA, USA). RNA integrity was assessed using the RNA Nano 6000 Assay Kit of the Bioanalyzer 2100 system (Agilent Technologies, Santa Clara, CA, USA).

#### 2.4.2. Library Preparation for Transcriptome Sequencing

A total amount of 5 μg RNA per sample was used as input material for the RNA sample preparation [50]. Sequencing libraries were generated using Illumina^®^ TruSeq^®^ Stranded Total RNA Sample Preparation kit. The first step involves the removal of ribosomal RNA (rRNA) with Ribo-Zero rRNA removal beads. The purification process is followed by, the fragments of RNA into small pieces. The cleaved RNA fragments are copied into the first cDNA strand using reverse transcriptase and random primers. Remaining overhangs were converted into blunt ends via exonuclease/polymerase activities. To select cDNA fragments of preferentially 150–200 bp in length the library fragments were purified with 2.0% Low Range Ultra Agarose. The products were enriched with PCR amplification using Phusion DNA polymerase (NEB) for 15 PCR cycles to create the final cDNA library. Library quality was assessed on the Agilent Bioanalyzer 2100 system.

The clustering of the index-coded samples was performed on a cBot Cluster Generation System using TruSeq PE Cluster Kit v3-cBot-HS (Illumina) according to the manufacturer’s instructions. After cluster generation the library preparations were sequenced on an Illumina Hiseq 2000 TruSeq SBS Kit v3-HS. An absolute log2 fold change ≥ 2.0 (*p* < 0.05) was used to determine differentially expressed genes.

#### 2.4.3. Reads Mapping to the Reference Genome

Raw data in fastq format was first processed by Trimmomatic with default parameters. Clean data was obtained by removing some unnecessary reads [51]. All downstream analyses were based on clean data of high quality. Reference genome and gene model annotation files were directly downloaded from the genome website with NCBI Reference Sequence: SZVX01000000. Index of the reference genome was built using Bowtie v2.0.6 while paired-end clean reads were aligned to the reference genome using TopHat v2.0.9 (Center for Bioinformatics and Computational Biology at the University of Maryland) [52]. We selected TopHat as the mapping tool as it can generate a database of splice junctions based on gene model annotation files. 

#### 2.4.4. Differential Expression Analysis and Functional Enrichment

The reads per kilobase of exon model per million reads (RPKM) of each gene was calculated according to the length of the gene and read counts mapped to this gene. RPKM considers the effect of sequencing depth and at the same time the gene length for the read count and is currently the most commonly used method for estimating gene expression levels. EdgeR (v 3.10, Bioconductor version) was used for differential expression analysis [53]. The DEGs between two samples were selected using the following criteria for significantly differential expression [50]: i) absolute value of log2 (fold change) was greater than 1 and ii) the false discovery rate (FDR) should be less than 0.05. To understand the functions of the differentially expressed genes GO functional enrichment and KEGG pathway analysis was carried out using Goatools and KOBAS respectively [54].

### 2.5. Enzymatic Hydrolysis

Strain WS52 was inoculated into 50 mL of various liquid medium with 1.0% or 5.0% of the different biomass and growth was observed for up to 4 days. Fermenting at 30 °C in the incubator with a rotate speed of 200 rpm, 1.0 mL of each sample was taken for enzymatic activity analysis every 24 h. Cultured cells were centrifuged at 15,000× *g* for 10 min and the supernatants were obtained. Sodium azide (0.05%) was added to prevent the microbial contamination during the hydrolysis [55]. All of the experiments were performed in triplicates. The results were expressed as mean ± standard deviation (SD) of at least three independent experiments. 

Enzymatic activities of CMCase were measured based on the microplate method [55] modified to 1ml volume with carboxymethyl cellulose as a substrate respectively. Briefly, 100 µL of diluted crude enzyme liquid was mixed with 200 µL 1.0% of various substance solution (pH 5.0) in 5ml test-tube, acting in the water bath at 50 °C for 30 min and instantly cooled down adding 600 µL of the DNS reagent and heated in boiling water for 5 min. 2000 µL water was added into each tube and take 200 µL mixed liquid for measuring the absorbance at 540 nm. The release amount of reducing sugar was calculated by the relevant standard curve.

The pectate lyase activity was also measured by the DNS reagent. In brief, 100 µL of supernatant sample was mixed with 900 µL of Pel reaction mix [100 mM Tris-HCl (pH 8.5), 0.5 mM CaCl2, and 0.5% polygalacturonic acid at 50 °C for 30 min.15 mg/mL cellulase and pectinase (Sigma) obtained from *Asperogillus niger* and *Rhizopus* sp. was used as control respectively.

### 2.6. Analytical Method of Monosaccharide Composition

Monosaccharide composition was analyzed using our previous method [56]. The resulting monosaccharides in the fermented broth were treated with the PMP derivation method and analyzed by HPLC (e2695, Waters, Arcade, NY, USA) on a Hypersil ODS-2(C18) column with UV detection. The monosaccharides were quantified using external calibration with an equimolar mixture of nine monosaccharide standards (arabinose, fucose, galactose, galacturonic acid, glucose, glucuronic acid, mannose, rhamnose, and xylose).

### 2.7. Accession Numbers of Draft Genome Sequencing and Transcription Sequencing, and Statistical Analyses

The Whole Genome Shotgun project has been deposited at DDBJ/ENA/GenBank under the accession SZVX00000000. The version described in this paper is version SZVX01000000. Reads from all sequencing experiments were deposited under accession numbers SRX5806498 for WS52-Tmt, SRX5806499 for WS52-Pep, and SRX5806500 for WS52-LB at the Sequence Read Archive: https://www.ncbi.nlm.nih.gov/sra. Statistical differences were determined using one-way analysis of variance (ANOVA) followed by Tukey’s test, considering *p* < 0.05 as significant, using SPSS version 17.0 (IBM Corp., Armonk, NY, USA). The results are presented as means ± standard deviation of three independent determinations.

## 3. Results and Discussion

Microbes are the cheapest and environmentally friendly substitutes for the pretreatment proper utilization of agrowaste and efficient biological conversion of plant stalk and wastes into bioproduct and bioenergy [57,58,59]. Thus, the isolation of microbial treasure for the digestion of sweet pepper and tomato stalk and decipher the genome, transcriptome and enzymes for the lignocellulolytic system has made this study vital. The following results and discussion have described the molecular insights of *Dickeya* sp. WS52 for the efficient digestion of Sweet Pepper and Tomato Stalk. 

### 3.1. Isolation and Identification of Plant Pathogen Dickeya sp. WS52

A plant-pathogen bacterium *Dickeya* sp. WS52 from soft-rotten tobacco plant (Figure S1). It showed strong ability to soften fresh *Solanaceae* vegetable stem and suggested that it may have the capacity to digest stalk biomass especially pectin. After initial digestion study of tomato and sweet pepper biomass, we found it can cause weight loss of biomass with 47.5% of vegetable after 4-day treatment. It was identified to belong to the genus *Dickeya* by 16S rDNA sequencing analysis (Genbank accession number MN689800). The strain WS52 was identified belonging to the species *Dickeya chrysanthemi* based on the value of the average nucleotide identity (ANI) and DDH (Table S1) [57].

### 3.2. Genomic analysis of Dickeya sp. WS52

The assembled genome of *Dickeya* sp. WS52 [GenBank: SZVX00000000] comprised a total of 4,744,455 bp containing 4082 protein coding sequences (CDS). To identify genes involved in lignocellulose degradation, CDS were analyzed using dbCAN carbohydrate-active enzymes (CAZy) annotation algorithm. Results indicated that the 122 genes have multiple domains assigned to CAZy families including 45 glycoside hydrolases (GHs), 14 carbohydrate esterases (CEs), 18 polysaccharide lyases (PLs), 4 enzymes with auxiliary activities (AAs) and 9 carbohydrate binding modules (CBMs) (Table 1).

To date, 71 *Dickeya* strains, 100 *Erwinia* strains, 148 *Pectobacterium* strains have been sequenced and deposited in the https://www.ncbi.nlm.nih.gov/genome/. To date, 17 *Dickeya* strains, 13 *Erwinia* strains and 29 *Pectobacterium* strains have been analyzed and deposited in the CAZy database (as of 17th of Sep 2019).

The genome of *Dickeya* sp. WS52 was compared with eight well-studied plant-pathogen bacteria: *D. Aquatica* 174/2 [GenBank: LT615367.1.], *D. chrysanthemi* Ech1591 [GenBank: NC_012912.1], *D. Dadantii* 3937 [GenBank: NC_014500 ], *D. fangzhongdai* PA1 [GenBank: NZ_CP020872.1], *D. solani* IFB0223 [GenBank: NZ_CP024710], *D. Zeae* Ech586 [GenBank: NC_013592.1], *Erwinia Amylovora* ATCC49946 [GenBank: NC_013971,NC_013972, NC_013973], *P ectobacterium carotovorum* SCC1 [GenBank: NZ_CP021894.1, NZ_CP021895.1], *Clostridium thermocellum* ATCC 27405 [GenBank: CP000568], *Clostridium bescii* DSM 6725 [GenBank:CP001393], *Sphingobium* sp. SYK-6 [GenBank:AP012222], *Enterobacter lignolyticus* SCF1 [GenBank: CP002272], *Pantoea ananatis* Sd-1 [GenBank:AZTE00000000].

The number of putative CAZy genes in *Dickeya* sp. WS52 was 122, including 45 GHs, 14 CEs 9 CBMs, 31 GTs, 18 PLs and 4 AAs. The percentage of putative CAZy genes (2.1%) was the highest in the compared genus *Dickeya* especially in the esterase family (Table 1). The amounts of CEs (14) in *Dickeya* sp. WS52 was significantly higher than those in most of the compared strains, except than that of CEs (16) *C. thermocellum* ATCC and of CEs (25) in *P. ananatis* Sd-1. The number of PLs (18) in *Dickeya* sp. WS52 which was equal to *D. chrysanthemi* Ech1591was the highest than those in all compared strains.

In the genome of WS52 there were 5 cellulase-encoding genes annotated to be cellulase: one endoglucanase (TYL41080.1, GH8) gene second cellulase (TYL43501.1, GH5) gene, third β-glucosidase genes (TYL43657.1, TYL43004.1, GH1; and TYL41537.1, GH3) (Table 2).

Besides, the cellulose enzymes there were comparable number of genes being annotated to be hemicellulose-degrading enzymes. There were four galactosidases including one alpha-galactosidase (TYL42149.1, GH36) two β-galactosidases (TYL42214.1, GH2; TYL44116.1, GH42) and one galactosidase (TYL44117.1, GH53). β-Galactosidase can degrade galactoside-containing cell wall polysaccharides and release free galactose. One α-L-rhamnosidase (TYL43305.1, GH78) and one xylan 1,3-β-xylosidase (TYL44137.1, GH43) were also found. There was one pair of glycogen debranching proteins related to glycogen degradation (TYL42790.1 glycogen debranching protein, TYL42791.1 and 1,4-alpha-glucan branching protein belonging to GH13 family) (Table 2). 

Activities in family GH28 were related to polygalacturonase, α-L-rhamnosidase, exo-polygalacturonase, exo-polygalacturonosidase, rhamnogalacturonase, rhamnogalacturonan α-1,2-galacturonohydrolase, and xylogalacturonan hydrolase which were almost involved into pectin digestion as intracellular enzymes or extracellular enzymes. In these four GH family proteins (TYL44351.1, 42856.1, 42857.1, and 42558.1 the former three enzymes were identified to be extracellular enzymes and TYL42558.1 as intracellular, suggesting these four enzymes played an important role in pectin digestion. Similarly, GH105 (TYL42562.1 and 42513.1) is often annotated to be unsaturated rhamnogalacturonyl hydrolase, d-4,5-unsaturated β-glucuronyl hydrolase, d-4,5-unsaturated α-galacturonidase. 

Activities in family GH1 were mainly related to β-glucosidase, β-galactosidase, β-mannosidase, β-glucuronidase, β-xylosidase, β-D-fucosidase et.al. All of the three enzymes (TYL42629.1, TYL42630.1 and TYL42639.1) were identified to be intracellular enzyme and might play avital role in the last step of polymer digestion.

Polysaccharide lyases is a group of enzymes that cleave uronic acid-containing polysaccharide chains via a β-elimination mechanism to generate an unsaturated hexenuronic acid residue and a new reducing end [21]. This section of the CAZy database presents a classification of these enzymes in families and subfamilies based on amino acid sequence similarities intended to reflect their structural features. Similar to other carbohydrate polymer degradation, enzymes related to pectin degradation would often secrete into extracellular environment and among the 18 polysaccharide lyases existed in the genome of WS52 only five proteins were identified to be intracellular enzymes which was mainly involved into the degradation of dimer or short polymers (Table 3).

Before the digestion of pectin by pectate lyases, deacetylation or esteration is vital to proceed the process [15]. Carbohydrate esterases catalyze the de-O or de-N-acylation of substituted saccharides. Therefore, we investigated the CE family in the genome of WS52 and found there were 14 esterase, half of which were extracellular enzymes, especially pectin esterase A (TYL43338.1, CE8), pectin esterase B (TYL41030.1, CE8) and pectin acetylesterase (TYL43339.1, CE12). There were six alpha/beta hydrolase (TYL43959.1, TYL43308.1, TYL40812.1, TYL40810.1, TYL41844.1, and TYL41326.1) belonging to CE10 was also annotated to be carboxylesterase, acetyl esterase or pectin acetyl-esterase (Table 4). TYL43659.1 was annotated to belong to carbohydrate acetyl esterase/feruloyl esterase which would not only be involved into lignin degradation, it can also cleave the ester bond between alcohol, oligosaccharide, polysaccharide and ferulic acid Therefore it can cut the complex cross-linking between polysaccharide and polysaccharide, and, polysaccharide and lignin, which will be helpful in the digestion of polysaccharide and the release of lignin. Feruloyl esterase forms a part of the enzyme complex that acts collectively and synergistically to completely hydrolyze xylan to its monomers. These enzymes have potential uses in the wide variety of applications of interest to the agri-food and pharmaceutical industries.

Several genes were annotated to be potentially involved in lignin degradation in *Dickeya* sp. WS52 (Table S5). These include multicopper oxidases (TYL43982.1 and TYL44660.1), catalase/hydroperoxidase (AA2), glucose-methanol-choline (GMC) family oxidoreductase (TYL44482.1, AA3), flavodoxin family protein (TYL43757.1 and TYL41583.1, AA6), glutathione S-transferases (GSTs), and quinone oxidoreductases. There were eight GST annotated to exist in the genome of *Dickeya* sp. WS52, which are TYL44756.1, TYL44033.1, TYL43409.1, TYL42345.1, TYL41649.1, TYL41540.1, TYL41013.1, TYL41021.1 and all of them functioned as intracellular proteins. NADH-quinone oxidoreductase was identified to be composed of thirteen subunits from TYL43983.1 to TYL43995.1. which were located between TYL43982.1 (PP52_00799) multicopper oxidase family protein and TYL43996.1 (PP52_00813) hydrogen peroxide-inducible genes activator. All of NADH-quinone oxidoreductase subunit were located as intracellular.

### 3.3. Transcriptal Profiling of Dickeya sp. WS52

#### 3.3.1. Identification of Expressed Transcripts and Expression Level Analysis in the WS52 Transcriptome

There were 3,973,361, 3,807,979 and 4,769,914 raw reads generated in sample LB, Pep and Tmt, respectively. After removing adapter and poly-N containing reads as well as low quality reads from raw data, 3,958,742, 3,801,205 and 4,754,608 clean reads were obtained and the error rate was 0.01% [51] (Table S2, Figure S2). The percentages of reads mapping to the reference genome were shown in Table S3. Among 4082 predicted protein-coding genes in the reference genome, 3154 (77.3% of the total genes, RPKM ≥ 15), 3029 (74.2% of the total genes, RPKM ≥ 15) and 3108 (76.1% of the total genes, RPKM ≥ 15) genes were detected in LB control, Pep and Tmt conditions, respectively (Table S4).

Further, the distribution of reads mapped to genomic regions were also investigated according gene structure, sequence coding for amino acids in protein (CDS) and intergenic regions. More than 80% of bases were mapped to CDS regions in LB sample, and 59.60% and 63.89% of bases were mapped to CDS regions in Pep and Tmt samples respectively (compared to 82.17% in LB samples) which indicated that Pep and Tmt samples have a similar response to vegetable stalk (Figure S4). Among these three samples, we did not find any difference from the chromosomal distributions of the reads in three samples, which suggest there was no significant region to be transcribed at the genome level (Figure S3).

#### 3.3.2. Identification of Differentially Expressed Genes Between Different Samples

**Figure 1:**
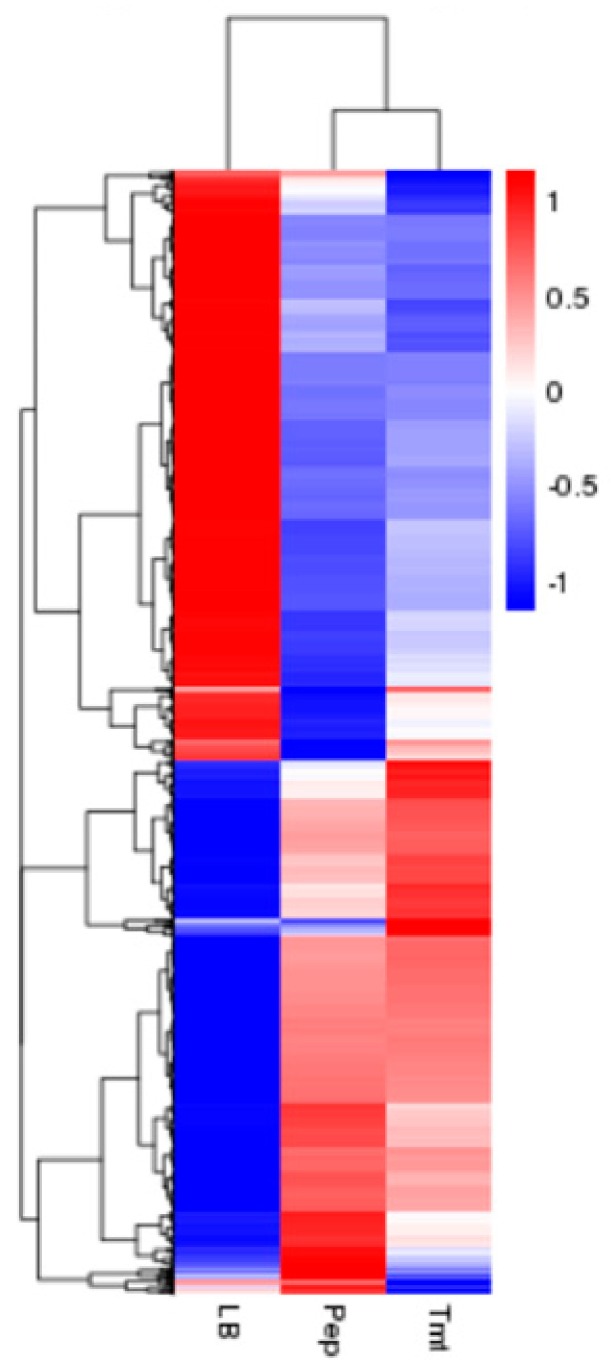
Cluster analysis of differentially expressed genes. the expression pattern in Pep and Tmt are similar compared to that in LB medium.

To understand the mechanism of digestion of vegetable stalk, differentially expressed genes (DEG) between different samples were analyzed by transcriptome studies. Comparison of the transcription levels of unigenes between vegetable stalk Pep and nutrient-rich sample LB revealed 1681 DEGs, including 767 upregulated genes and 914 downregulated genes (Figure 1 and Figure S7A). Comparison of vegetable stalk Tmt and LB identified 1574 DEGs, which included 744 upregulated and 830 downregulated genes (Figure S7B). Comparison between these two vegetable stalks displayed 209 DEGs, of which 95 were upregulated and 114 downregulated (Figure S7C). The total number of DEGs was also shown in a Venn diagram (Figure S8).

#### 3.3.3. Functional Distribution of Differentially Expressed Genes

**Figure 2:**
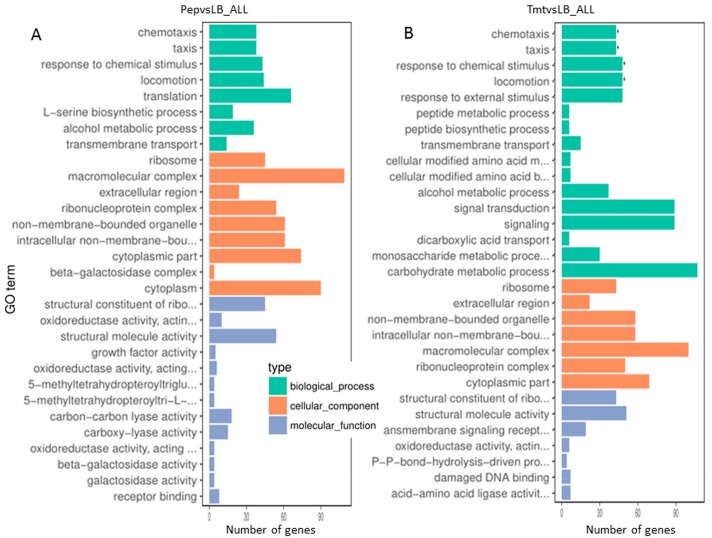
Comparing functional annotations between different samples (**A**) Comparable group: Pep and LB; (**B**) Comparable group: Tmt and LB; The green bars represent biological process; orange bars represent cellular component; purple bars represent molecular function.

Based on GO (Gene ontology) categories, we found some major functional groups were significantly upregulated in Tmt vs LB, which included chemotaxis, taxis, response to chemical stimulus and locomotion (Figure 2B). Besides the mentioned above, we found beta-galactosidase complex in type cellular component, beta-galactosidase activity, galactosidase activity in molecular function were listed in the most enriched GO terms (Pep vs LB) (Figure 2A). However, no group was significantly upregulated (Figure 2A).

**Figure 3:**
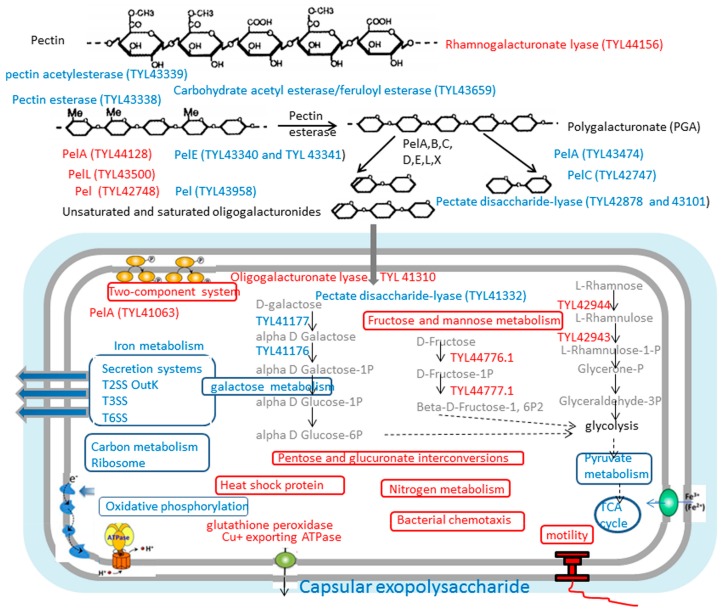
Overview of pathways and functions affected by the vegetable stalk in MSM medium compared to LB medium. Functions marked in red are upregulated while those in blue are downregulated.

KEGG pathway enrichment was also analyzed [58], the upregulated top major pathways in Pep vs LB and/or Tmt vs LB were two-component system, bacterial chemotaxis, flagellar assembly, nitrogen metabolism, arginine biosynthesis, folate biosynthesis, fructose, and mannose metabolism et. al. on the contrary, the downregulated pathways were mainly related to bacterial secretion system, citrate cycle (TCA cycle), carbon metabolism, pyruvate metabolism, ribosome, pentose and glucuronate interconversions, glycolysis/gluconeogenesis, oxidative phosphorylation, microbial metabolism in diverse environments, galactose metabolism et. al. (Figure 3)

The fructose and mannose metabolism pathway contained the glycolysis of L-rhamnose and D-fructose, we found the first two enzymes during the glycolysis of the two sugars were significantly upregulated (Figure 3). Log2FC of the mRNA related with L-rhamnose isomerase (TYL42944.1) reached 4.04 and 4.80 in PepvsLB and TmtvsLB, respectively, and those of rhamnulokinase (TYL42943.1) reached 3.30 and 3.67 in PepvsLB and TmtvsLB, respectively (Table 5). During the glycolysis of D-fructose, the first two enzymes, PTS fructose transporter subunit IIBC (TYL44777.1) and 1-phosphofructokinase (TYL44776.1) in the pathway were also significantly upregulated. On the contrary, the pathway of galactose metabolism was significantly downregulated. The transcription level related to galactose mutarotase (TYL41177.1) reached −1.39 and −1.63 and those of galactokinase (TYL41176.1) reached −2.21 and −2.45, in PepvsLB and TmtvsLB, respectively (Table 5).

#### 3.3.4. Most Enzymes Related to Carbohydrate Metabolism Were Downregulated under the Vegetable Stalk

We analyzed the expression of plant cell wall degrading enzymes (Table 2, Table 3 and Table 4) especially, we analyzed the transcriptional level of pectin-degrading-related enzymes. Most pectinase genes were significantly downregulated in the nutrient-poor medium Pep and Tmt during stationary phase except PelA (TYL44128), PelL (TYL43500, 1.30 in Pep sample only), PelC (TYL42748.1, 1.11 in Pep sample only), and rhamnogalacturonate lyase (TYL44156, 2.57 and 2.08) which may act more specifically on certain pectin glycans. 

There were three PelA genes annotated in genome of WS52 (Table 3), PelA (TYL43474) and PelA (TYL44128) were identified to extracellular protein. The former was downregulated, and the latter was upregulated in Pep and Tmt sample. Furthermore, PelA (TYL41063) was upregulated as intracellular protein. The most important is that we found that the extracellular enzymes related to the pretreatment of pectin were all downregulated in vegetable stalks, for example, pectin acetyl-esterase (TYL43339, −1.86 and −1.47), pectin esterase (TYL43338, −1.02 and −2.24) and Carbohydrate acetyl esterase/feruloyl esterase (TYL43659.1, −2.28 and −2.07).

After digestion of pectin in extracellular environment, oligogalacturonate would be transferred into cells and would be cut by significantly upregulated intracellular enzymes oligogalacturonate lyase (TYL41310.1, 3.05 and 1.50) (Table 3 and Figure 3). As shown in Figure 3, TYL41063.1 (pectate lyase PelA), TYL41310.1 (oligogalacturonate lyase), and TYL41332.1 (pectate disaccharide-lyase) were also further identified not to be secreted by Secretome P 2.0 Server. Some GH28 and GH19 family was significantly upregulated (Table 2) However, most galactosidase and beta-glucosidase were downregulated. For example, β-glucosidase (TYL43657.1, −6.76 and −6.38) involved in the cellulose digestion.

### 3.4. Growth and Enzymatic Profiling of Dickeya sp. WS52 in Sweet Pepper and Tomato Stalk Medium

**Figure 4:**
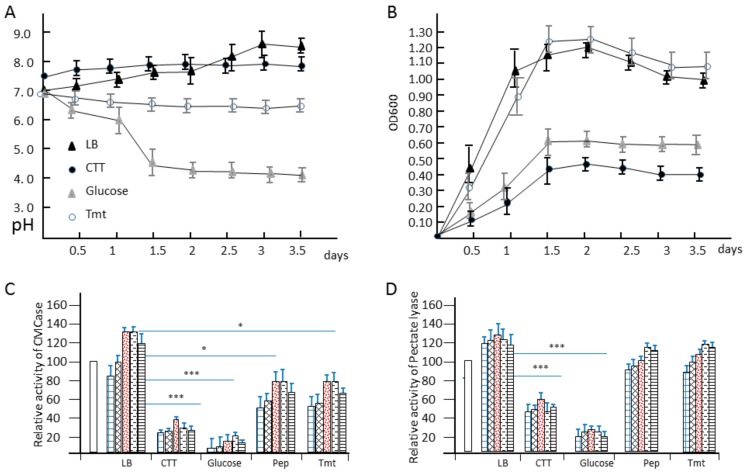
The growth and enzymatic activity of strain WS52 in different medium. (**A**) the pH change was measured in different medium; (**B**) the growth curve of strain WS52 were shown in OD600; (**C**) the CMCase activity were shown in relative activity in different medium; (**D**) the pectate lyase activity were shown in relative activity in different medium. Three asterisks indicate *p* < 0.01, one asterisk indicates *p* < 0.05.

*Dickeya* sp., plant pathogen bacteria in soil, can have different growth style and secret different enzymes before and after infection to plant issues. To understand the relationship between medium and enzyme activity, we cultured this strain in four different mediums and investigate the enzymatic profiling. Firstly we focused on the growth speed and the change of pH during the cultivation (Figure 4A,B). The growth pattern of WS52 in LB is similar to that in MM + 1.0% vegetable stalks, where sweet pepper or tomato stalk was used. However, on the first day the concentration of bacteria cells in LB medium was higher than that in vegetable stalks. After 1.5 days, the OD600 value in vegetable stalk is higher than that in LB medium and reached the highest point 1.20 on the second day. The growth rate in MM + glucose was slow, and reached the stationary phase at the OD600 value 0.55 on 1.5 day. The grow pattern in CTT medium was similar to that in MM + Glucose. However, OD600 value only reached the highest point 0.40. Simultaneously, we also investigate the changed of pH in these four media. We found the pH in MM + glucose changed hugely, at the first day the pH changed from 7.0 to 6.0 and then decrease sharply to 4.5 on 1.5 day. The original pH in LB medium and vegetable stalks was 7.0, but the pH change pattern was oppositely, the pH in LB medium steadily increased up to the highest point 8.3 at the third day. On the contrary, there was only slight decrease in vegetable stalks, and it reached pH 6.8. The pH in CTT medium was not changed hugely, from 7.6 to 8.0.

To investigate the shift of enzyme activity in these culture conditions, we took the 1ml broth at 1, 1.5, 2, 2.5, 3 days in these four media (Figure 4C,D). We used 15 mg/mL commercial cellulase and pectinase from sigma as control. We found the enzymatic activity of CMCase and pectinase in LB medium was highest, and the relative activity reached 135 ± 5% in CMCase and 130 ± 4% in pectinase at 2 days and 2.5 days. The activity in MM + vegetable stalks was the second highest. The relative activity of CMCase only reached 81 ± 6% at 2 days and 2.5 days and 110 ± 7% in pectinase at 2.5 days and 3 days. The relative activity of CMCase in CTT medium and MM + glucose was slow, reached less than 40% and 20%, respectively. However, the relative activity of pectinase can reach 60% and 30%. These results indicated that only addition of vegetable stalks in MSM medium did not effectively induce the expression of lignocellulose-degrading enzymes, which was consistent the result of transcriptional sequencing.

### 3.5. The Component Analysis of Monosaccharide and the Transcriptional Level of Sugar Glycolysis of Dickeya sp. WS52

**Figure 5:**
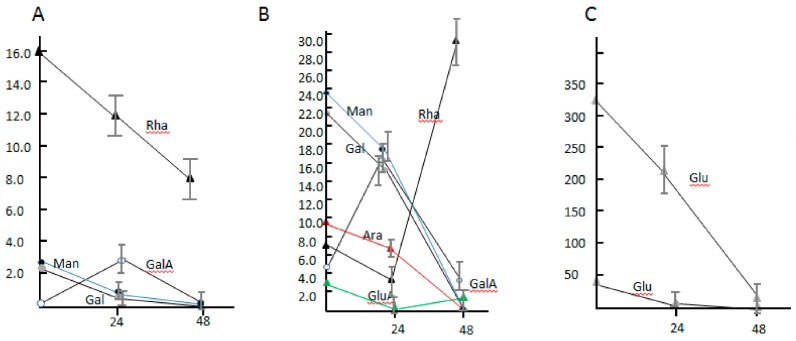
The monosaccharide component shift in Minimal Salt Medium (MSM) + vegetable stalks. (**A**) monosaccharide component shift in 1% MSM + vegetable stalks except glucose; (**B**) monosaccharide component shift in 5% MSM + vegetable stalks except glucose; (**C**) the change of glucose concentration in 1% or 5% MSM + vegetable stalks.

To understand the total effect of lignocellulose degrading enzymes secreted by strain WS52 on the vegetable stalk, we measured the component of monosaccharide in the broth of 1.0% and 5.0% tomato stalk in MSM medium for two days at 30 °C (Figure 5). The changes of component of monosaccharide not only reflected the secretion and activity of mixed enzymes, but also reflected the ability of glycolysis of monosaccharide in the cells of WS52. In the 1.0% vegetable stalk medium, mannose, galactose, and glucose were gradually consumed from the original concentration, 2.52 ± 0.72 μg/mL, 2.37 ± 0.54 μg/mL, 46.72 ± 5.68 μg/mL during the two-days fermentation, respectively (Figure 5A,C). The concentration of rhamnose decreased from 15.94 ± 1.48 μg/mL to 12.35 ± 1.65 μg/mL in the first day, 7.63 ± 1.05 μg/mL in the second day. Interestingly, galacturonic acid, the product of pectin digestion, was not detected originally in the second days, and it was only detected to be 2.23 ± 0.38 μg/mL in the first day, indicating it was produced by the secreted pectin-degraded enzymes and consumed via glycolysis. 

In 5.0% vegetable stalk medium, the change pattern of mannose, galactose, and glucose was similar to that in 1.0% vegetable stalk medium (Figure 5B). Arabinose was gradually consumed from 9.13 ± 1.25 μg/mL to 6.66 ± 1.01 μg/mL in the first day to zero in the second day, which was not detected during fermentation of the 1.0% vegetable stalk medium. The shift of galacturonic acid was similar to that in 1% vegetable stalk, from 5.5 ± 0.61 μg/mL originally to 17.19 ± 2.01 μg/mL in the first day to 3.63 ± 0.74 μg/mL in the second day. Glucuronic acid was detected, and the concentration was shown from decrease to increase, that was similar to rhamnose maybe indicating as consume of sugar or growth of cells proceeded, the secreted enzyme digested the pectin, for example, the significant upregulated rhamnogalacturonate lyase, the concentration of rhamnose increased accordingly.

## 4. Conclusions

The plant pathogen *Dickeya* sp. WS52 with strong pectin degradation capacity has been isolated from soft-rotten tobacco. Based on the draft genome, we performed the comprehensive analysis of its CAZy degradative system and found a complete repertoire of enzymes required for hemicellulose degradation, especially pectinases and pectin esterases, more than other *Dickeya* species. Using MSM liquid medium supplemented with sweet pepper and tomato vegetable stalks, we performed the transcriptome sequencing to investigate their expression pattern compared with nutrient-rich LB medium, revealing that parts of genes encoding lignocellulolytic enzymes were significantly upregulated. However, most genes related to lignocellulolytic enzymes were downregulated due to the slow growth and downregulated secretion systems. Furthermore, considering the enzyme activity in MSM + vegetable stalks, we found the enzymes activity was not higher that in LB medium, suggesting *Dickeya* sp. WS52 would need sufficient nutrient to express and to improve secretion system. As a plant pathogen, it may not be useful to make fertilizer for agriculture. However, this identified degradative system is valuable for the lignocellulosic bioenergy industry and animal production. 

## Figures and Tables

**Table 1:** Comparison of genome of *Dickeya* sp. WS52 with other pectin degrading strains or plant pathogen bacteria.

Species	Genome Size (bp)	Protein Coding Genes	% CAZy	GHs	CEs	CBMs	PLs
*Dickeya* sp. WS52	4,744,455	4082	2.1	45	14	9	18
*D. Aquatica* 174/2	4,501,560	4079	1.8	38	6	12	16
*D. chrysanthemi* Ech1591	4,813,854	4112	1.8	42	6	8	18
*D. Dadantii* 3937	4,922,802	4244	1.9	46	6	9	17
*D. fangzhongdai* DSM101947	503,245	4298	1.8	43	6	11	17
*D. fangzhongdai* PA1	4,979,223	4208	1.8	42	6	13	16
*D. solani* IFB0223	4,937,554	4143	1.8	42	6	12	16
*D. Zeae* Ech586	4,818,394	4113	1.7	41	5	8	14
*E. Amylovora* ATCC49946	3,905,604	3488	1.4	36	3	8	2
*P. carotovorum* SCC1	4,980,322	4294	1.9	47	6	13	15
*C. thermocellum* ATCC27405	3,843,300	3263	5.6	74	16	90	4
*C. bescii* DSM6725	2,931,660	2662	2.9	49	6	18	3
*Sphingobium* sp. SYK-6	4,348,130	3939	0.7	21	1	3	0
*E. lignolyticus* SCF1	4,814,050	4350	1.2	42	5	7	0
*P. ananatis* Sd-1	4,927,500	4332	2.2	59	25	11	2

Abbreviations: % CAZy: Percentage of listed CAZy genes in protein coding genes; GHs: glycoside hydrolase family; CEs: carbohydrate esteraseses; CBMs: carbohydrate binding modules; PLs: polysaccharide lyases.

**Table 2:** Cellulose and hemicellulose degradation relevant enzymes and its transcriptome in sweet pepper or tomato compared to LB medium: glycoside hydrolase family protein.

ORF Number (TYL)	Annotation	GH Family	AA Length	Pepper	Tomato	SignalP
43657.1	β-glucosidase	1	429	−6.76	−6.38	N
43004.1	β-glucosidase	1	429	−1.57	n.d.	N
42629.1	GH 1 protein	1	429	−3.28	−3.24	N
42630.1	GH 1 protein	1	429	−2.91	−3.63	N
42639.1	GH 1 protein	1	429	n.d.	n.d.	N
42214.1	β-galactosidase	2	752	−1.22	−1.39	N
43595.1	beta-N-acehexosaminidase	3	216	n.d.	n.d.	N
41537.1	β-glucosidase	3	216	−2.50	−1.80	Y
41574.1	GH 3 protein	3	216	n.d.	n.d.	Y
43501.1	cellulase family glycosylhydrolase	5	275	n.d.	−1.44	N
41080.1	endoglucanase	8	320	n.d.	1.08	Y
42790.1	glycogen debranching protein	13	299	n.d.	n.d.	N
42791.1	1,4-alpha-glucan branching protein	13	299	n.d.	n.d.	N
42742.1	GH 19 protein	19	231	3.87	5.04	N
44181.1	lytic murein transglycosylase	23	135	−1.04	n.d.	Y
43265.1	murein transglycosylase D	23	135	1.22	1.53	N
43333.1	lytic murein transglycosylase	23	135	n.d.	n.d.	N
42383.1	murein transglycosylase	23	135	1.01	1.03	Y
43901.1	lysozyme	24	137	n.d.	n.d.	N
44351.1	GH 28 protein	28	325	n.d.	n.d.	Y
42856.1	GH 28 protein	28	325	n.d.	1.18	Y
42857.1	GH 28 protein	28	325	1.10	n.d.	Y
42558.1	GH 28 protein	28	325	n.d.	n.d.	N
43601.1	GH 31 protein	31	427	1.58	1.73	N
43027.1	sucrose-6-phosphate hydrolase	32	293	n.d.	n.d.	N
42500.1	glycosyl hydrolase	33	342	n.d.	n.d.	N
42149.1	alpha-galactosidase	36	688	−4.26	−4.57	N
44116.1	β-galactosidase	42	371	−1.23	−1.57	N
44137.1	Xylan 1,3-β-xylosidase	43	248	n.d.	−1.05	Y
41284.1	GH43 protein	43	248	n.d.	n.d.	N
44117.1	galactosidase	53	342	−2.27	−2.16	Y
43528.1	peptidoglycan hydrolase	73	128	n.d.	n.d.	N
41480.1	hypothetical protein	73	128	n.d.	n.d.	N
42781.1	4-alpha-glucanotransferase	77	494	n.d.	n.d.	N
43305.1	alpha-L-rhamnosidase	78	504	n.d.	n.d.	N
44201.1	murein transglycosylase A	102	157	n.d.	n.d.	N
41622.1	lytic murein transglycosylase	103	295	1.68	1.74	Y
41246.1	lytic murein transglycosylase B	103	295	−1.88	−1.67	N
43711.1	GH 104 protein	104	145	n.d.	n.d.	N
43498.1	GH 104 protein	104	145	n.d.	n.d.	N
42562.1	GH 105 protein	105	332	n.d.	n.d.	N
42513.1	GH 105 protein	105	332	−4.96	−3.73	N
44519.1	Oxidoreductase	109	126	n.d.	n.d.	N
44637.1	Gfo/Idh/MocA family-oxidoreductase	109	126	−1.33	−1.20	N

n.d.: Not Differentially expressed gene.

**Table 3:** Pectin degradation relevant polysaccharide lyases (PL) enzymes and its transcriptome profile in pepper or tomato stalk compared to Luria Bertani (LB) medium.

ORF Number (TYL)	Annotation	PL	AA Length	Log2FC in Pep	Log2FC in Tmt	SignalP
43957.1	hypothetical protein	1	202	2.14	1.94	N
43340.1	Pectate lyase E	1	202	n.d.	−1.85	Y
43341.1	Pectate lyase E	1	202	−4.21	−4.24	Y
43474.1	Pectate lyase A	1	202	n.d.	−1.83	Y
42747.1	Pectate lyase C	1	202	n.d.	−1.33	Y
42748.1	Pectate lyase C	1	202	1.11	n.d.	Y
42878.1	Pectate disaccharide-lyase	1	202	−1.68	−2.31	Y
41268.1	Pectin lyase	1	202	n.d.	n.d.	N
41332.1	Pectate disaccharide-lyase	2	530	−1.04	−1.81	N
44128.1	Pectate lyase A	3	197	1.75	n.d.	Y
41063.1	Pectate lyase A	3	197	n.d.	1.73	N
44527.1	Rhamnogalacturonate lyase	4	567	n.d.	n.d.	Y
44156.1	Rhamnogalacturonate lyase	4	567	2.57	2.08	Y
43101.1	Pectate disaccharide-lyase	9	374	−1.33	−2.13	Y
43500.1	Pectate lyase L	9	374	1.30	n.d.	Y
44668.1	Pectate lyase L	9	374	n.d.	n.d.	Y
43958.1	pectate lyase	10	287	−3.92	−4.85	Y
41310.1	Oligogalacturonate lyase	22	265	3.05	1.50	N

n.d.: Not Differentially expressed gene.

**Table 4:** Pectin degradation relevant CE enzymes and its transcriptome profile in pepper or tomato stalk compared to LB medium.

ORF Number (TYL)	Annotation	CE	AA Length	Log2FC in Pep	Log2FC in Tmt	SignalP
44765.1	S-formylglutathione hydrolase	1	227	−2.63	−2.26	N
44076.1	enterochelin esterase	1	227	n.d.	n.d.	N
43659.1	Carbohydrate acetyl esterase/feruloyl esterase	1	227	−2.28	−2.07	Y
43338.1	Pectin esterase A	8	288	−1.02	−2.24	Y
41030.1	Pectin esterase B	8	288	n.d.	n.d.	Y
44436.1	N-acetylglucosamine-6-phosphate deacetylase	9	373	n.d.	n.d.	N
43959.1	alpha/beta hydrolase	10	341	n.d.	2.15	N
43308.1	alpha/beta hydrolase	10	341	−2.60	−1.67	Y
40812.1	alpha/beta hydrolase	10	341	n.d.	n.d.	Y
40810.1	alpha/beta hydrolase	10	341	n.d.	−1.28	N
41844.1	alpha/beta hydrolase	10	341	n.d.	n.d.	Y
41326.1	alpha/beta hydrolase	10	341	−1.62	−1.87	N
42473.1	N-acetylglucosamine deacetylase	11	271	1.09	n.d.	N
43339.1	pectin acetylesterase	12	210	−1.86	−1.47	Y

n.d.: Not Differentially expressed gene.

**Table 5:** Potential sugar metabolism relevant enzymes and its transcriptome profile in pepper or tomato stalk compared to LB medium.

ORF Number (TYL)	Log2FC in Pep	Log2FC in Tmt	Annotation
44776.1	2.26	2.38	PTS fructose transporter subunit IIBC
44777.1	2.90	3.02	1-phosphofructokinase
44778.1	1.47	1.27	fused PTS fructose transporter subunit IIA
42943.1	3.30	3.67	rhamnulokinase
42944.1	4.04	4.80	L-rhamnose isomerase
42946.1	4.02	3.74	L-rhamnose mutarotase
44512.1	n.d.	n.d.	mannose-6-phosphate isomerase
44525.1	n.d.	n.d.	L-arabinose isomerase
43854.1	n.d.	n.d.	mannose-6-phosphate isomerase
41342.1	1.17	1.68	PTS mannose transporter subunit IID
41344.1	n.d.	n.d.	PTS mannose transporter subunit IIAB
44504.1	1.20	1.48	mannosyl-3-phosphoglycerate
42336.1	n.d.	n.d.	glucuronate isomerase
41873.1	n.d.	n.d.	glucose-6-phosphate isomerase
41752.1	−1.84	n.d.	arabinose-5-phosphate isomerase KdsD
43476.1	−1.28	−1.30	xylose isomerase
43187.1	−1.72	−2.11	xylose isomerase
41176.1	−2.21	−2.45	galactokinase
41177.1	−1.39	−1.63	galactose mutarotase

n.d.: Not Differentially expressed gene.

## Supplementary Materials

The following are available online at https://www.mdpi.com/2218-273X/9/12/753/s1, Figure S1: the surface symptom (Figure S1A) and the inner symptom (Figure S1B) of soft-rotten tobacco plant infected by *Dickeya* sp. WS52, Figure S2: Classification of Raw Reads of three samples, Figure S3: Reads density in chromosome of three samples, Figure S4: Distribution of reads mapped to genomic regions of three samples, Figure S5: FPKM distribution for all samples, Figure S6: The correlation between samples, Figure S7: the overall distribution of DEGs in three samples, Figure S8: Comparisons of the number and overlapping relationships of DEGs between different samples, Figure S9 The most enriched GO terms between Pep and Tmt, Table S1: The identification of strain WS52 based on ANI, Table S2: Transcriptome data output of three samples, Table S3: Percentages of genes in different expression levels, Table S4: Percentages of reads mapping to the reference genome, Table S5: The transcriptional level of potentially lignin degradation related genes in MSM + vegetable stalks compared to LB medium, Table S6: The transcriptome of ribosomal proteins in MSM + pepper or tomato stalk compared to LB medium, Table S7: The transcriptome of flagellar-forming proteins in MSM + pepper or tomato stalk compared to LB medium, Table S8: The transcriptome related with capsular in MSM + pepper or tomato stalk compared to LB medium, Table S9: The transcriptome of type II secretion system protein in MSM + pepper or tomato stalk compared to LB medium, Table S10: The transcriptome of type III secretion system protein in MSM + pepper or tomato stalk compared to LB medium, Table S11: The transcriptome of type VI secretion system protein in MSM + pepper or tomato stalk compared to LB medium.

## References

[1] Badhan A., Ribeiro G.O., Jones D.R., Wang Y., Abbott D.W., Di Falco M., Tsang A., McAllister T.A. (2018). Identification of novel enzymes to enhance the ruminal digestion of barley straw. Bioresour. Technol..

[2] Wang T., Zhang R., Su W., Lu Q., Dong C. (2016). Study on pyrolysis characteristics of red pepper stalks to analyze the changes of pyrolytic behaviors from xylophyta to herbage. J. Anal. Appl. Pyrolysis.

[3] Kim S., Dale B.E. (2004). Global potential bioethanol production from wasted crops and crop residues. Biomass Bioenergy.

[4] Xiong X.Q., Liao H.D., Ma J.S., Liu X.M., Zhang L.Y., Shi X.W., Yang X.L., Lu X.N., Zhu Y.H. (2014). Isolation of a rice endophytic bacterium, Pantoea sp. Sd-1, with ligninolytic activity and characterization of its rice straw degradation ability. Lett. Appl. Microbiol..

[5] Wei L., Shutao W., Jin Z., Tong X. (2014). Biochar influences the microbial community structure during tomato stalk composting with chicken manure. Bioresour. Technol..

[6] Zhang G., Li S., Xu Y., Wang J., Wang F., Xin Y., Shen Z., Zhang H., Ma M., Liu H. (2019). Production of alkaline pectinase: A case study investigating the use of tobacco stalk with the newly isolated strain Bacillus tequilensis CAS-MEI-2-33. BMC Biotechnol..

[7] Farias N., Almeida I., Meneses C. (2018). New Bacterial Phytase through Metagenomic Prospection. Molecules.

[8] Meneses C., Silva B., Medeiros B., Serrato R., Johnston-Monje D. (2016). A Metagenomic Advance for the Cloning and Characterization of a Cellulase from Red Rice Crop Residues. Molecules.

[9] Mohnen D. (2008). Pectin structure and biosynthesis. Curr. Opin. Plant Biol..

[10] Nakkeeran E., Umesh-Kumar S., Subramanian R. (2011). Aspergillus carbonarius polygalacturonases purified by integrated membrane process and affinity precipitation for apple juice production. Bioresour. Technol..

[11] Caffall K.H., Mohnen D. (2009). The structure, function, and biosynthesis of plant cell wall pectic polysaccharides. Carbohydr. Res..

[12] Vincken J.P., Schols H.A., Oomen R.J., McCann M.C., Ulvskov P., Voragen A.G., Visser R.G. (2003). If homogalacturonan were a side chain of rhamnogalacturonan I. Implications for cell wall architecture. Plant Physiol..

[13] Broxterman S.E., Schols H.A. (2018). Characterisation of pectin-xylan complexes in tomato primary plant cell walls. Carbohydr. Polym..

[14] Latarullo M.B., Tavares E.Q., Maldonado G.P., Leite D.C., Buckeridge M.S. (2016). Pectins, Endopolygalacturonases, and Bioenergy. Front. Plant Sci..

[15] Amin F., Bhatti H.N., Bilal M. (2019). Recent advances in the production strategies of microbial pectinases—A review. Int. J. Biol. Macromol..

[16] Christgau S., Kofod L.V., Halkier T., Andersen L.N., Hockauf M., Dorreich K., Dalboge H., Kauppinen S. (1996). Pectin methyl esterase from Aspergillus aculeatus: Expression cloning in yeast and characterization of the recombinant enzyme. Biochem. J..

[17] Li Q., Coffman A.M., Ju L.K. (2015). Development of reproducible assays for polygalacturonase and pectinase. Enzym. Microb. Technol..

[18] Hoondal G.S., Tiwari R.P., Tewari R., Dahiya N., Beg Q.K. (2002). Microbial alkaline pectinases and their industrial applications: A review. Appl. Microbiol. Biotechnol..

[19] Yim S.S., Choi J.W., Lee S.H., Jeong K.J. (2016). Modular Optimization of a Hemicellulose-Utilizing Pathway in Corynebacterium glutamicum for Consolidated Bioprocessing of Hemicellulosic Biomass. ACS Synth. Biol..

[20] Kashyap D.R., Vohra P.K., Chopra S., Tewari R. (2001). Applications of pectinases in the commercial sector: A review. Bioresour. Technol..

[21] Cantarel B.L., Coutinho P.M., Rancurel C., Bernard T., Lombard V., Henrissat B. (2009). The Carbohydrate-Active EnZymes database (CAZy): An expert resource for Glycogenomics. Nucleic Acids Res..

[22] Lombard V., Bernard T., Rancurel C., Brumer H., Coutinho P.M., Henrissat B. (2010). A hierarchical classification of polysaccharide lyases for glycogenomics. Biochem. J..

[23] Garron M.L., Cygler M. (2010). Structural and mechanistic classification of uronic acid-containing polysaccharide lyases. Glycobiology.

[24] Nakkeeran E., Subramanian R., Umesh Kumar S. (2008). Improving specific activity of Aspergillus carbonarius polygalacturonase using polymeric membranes. Appl. Biochem. Biotechnol..

[25] Busto M.D., Garcia-Tramontin K.E., Ortega N., Perez-Mateos M. (2006). Preparation and properties of an immobilized pectinlyase for the treatment of fruit juices. Bioresour. Technol..

[26] Bajpai P. (1999). Application of enzymes in the pulp and paper industry. Biotechnol. Prog..

[27] Omogbenigun F.O., Nyachoti C.M., Slominski B.A. (2004). Dietary supplementation with multienzyme preparations improves nutrient utilization and growth performance in weaned pigs. J. Anim. Sci..

[28] Tang Y., Wu P., Jiang S., Selvaraj J.N., Yang S., Zhang G. (2019). A new cold-active and alkaline pectate lyase from Antarctic bacterium with high catalytic efficiency. Appl. Microbiol. Biotechnol..

[29] Bonavita A., Carratore V., Ciardiello M.A., Giovane A., Servillo L., D’Avino R. (2016). Influence of pH on the Structure and Function of Kiwi Pectin Methylesterase Inhibitor. J. Agric. Food Chem..

[30] Cheirsilp B., Umsakul K. (2008). Processing of banana-based wine product using pectinase and alpha-amylase. J. Food Process. Eng..

[31] Alencar B.R.A., Dutra E.D., Sampaio E., Menezes R.S.C., Morais M.A. (2018). Enzymatic hydrolysis of cactus pear varieties with high solids loading for bioethanol production. Bioresour. Technol..

[32] Hugouvieux-Cotte-Pattat N., Condemine G., Shevchik V.E. (2014). Bacterial pectate lyases, structural and functional diversity. Environ. Microbiol. Rep..

[33] Laothanachareon T., Bunterngsook B., Suwannarangsee S., Eurwilaichitr L., Champreda V. (2015). Synergistic action of recombinant accessory hemicellulolytic and pectinolytic enzymes to Trichoderma reesei cellulase on rice straw degradation. Bioresour. Technol..

[34] Wang J., Chio C., Chen X., Su E., Cao F., Jin Y., Qin W. (2019). Efficient saccharification of agave biomass using Aspergillus niger produced low-cost enzyme cocktail with hyperactive pectinase activity. Bioresour. Technol..

[35] Kobayashi T., Hatada Y., Higaki N., Lusterio D.D., Ozawa T., Koike K., Kawai S., Ito S. (1999). Enzymatic properties and deduced amino acid sequence of a high-alkaline pectate lyase from an alkaliphilic Bacillus isolate. Biochim. Biophys. Acta.

[36] Blanco P., Sieiro C., Villa T.G. (1999). Production of pectic enzymes in yeasts. FEMS Microbiol. Lett..

[37] Blandino A., Iqbalsyah T., Pandiella S.S., Cantero D., Webb C. (2002). Polygalacturonase production by Aspergillus awamori on wheat in solid-state fermentation. Appl. Microbiol. Biotechnol..

[38] Bruhlmann F. (1995). Purification and characterization of an extracellular pectate lyase from an Amycolata sp.. Appl. Environ. Microbiol..

[39] Reverchon S., Nasser W. (2013). Dickeya ecology, environment sensing and regulation of virulence programme. Environ. Microbiol. Rep..

[40] Wormit A., Usadel B. (2018). The Multifaceted Role of Pectin Methylesterase Inhibitors (PMEIs). Int. J. Mol. Sci..

[41] Kazemi-Pour N., Condemine G., Hugouvieux-Cotte-Pattat N. (2004). The secretome of the plant pathogenic bacterium Erwinia chrysanthemi. Proteomics.

[42] Nivaskumar M., Francetic O. (2014). Type II secretion system: A magic beanstalk or a protein escalator. Biochim. Biophys. Acta.

[43] Jiang X., Zghidi-Abouzid O., Oger-Desfeux C., Hommais F., Greliche N., Muskhelishvili G., Nasser W., Reverchon S. (2016). Global transcriptional response of Dickeya dadantii to environmental stimuli relevant to the plant infection. Environ. Microbiol..

[44] Reverchon S., Robert-Baudouy J. (1987). Regulation of expression of pectate lyase genes pelA, pelD, and pelE in Erwinia chrysanthemi. J. Bacteriol..

[45] Tardy F., Nasser W., Robert-Baudouy J., Hugouvieux-Cotte-Pattat N. (1997). Comparative analysis of the five major Erwinia chrysanthemi pectate lyases: Enzyme characteristics and potential inhibitors. J. Bacteriol..

[46] Lowe-Power T.M., Hendrich C.G., von Roepenack-Lahaye E., Li B., Wu D., Mitra R., Dalsing B.L., Ricca P., Naidoo J., Cook D. (2018). Metabolomics of tomato xylem sap during bacterial wilt reveals Ralstonia solanacearum produces abundant putrescine, a metabolite that accelerates wilt disease. Environ. Microbiol..

[47] Raoul des Essarts Y., Pedron J., Blin P., Van Dijk E., Faure D., Van Gijsegem F. (2019). Common and distinctive adaptive traits expressed in Dickeya dianthicola and Dickeya solani pathogens when exploiting potato plant host. Environ. Microbiol..

[48] Fahy A., McGenity T.J., Timmis K.N., Ball A.S. (2006). Heterogeneous aerobic benzene-degrading communities in oxygen-depleted groundwaters. FEMS Microbiol. Ecol..

[49] Yoon S.H., Ha S.M., Kwon S., Lim J., Kim Y., Seo H., Chun J. (2017). Introducing EzBioCloud: A taxonomically united database of 16S rRNA gene sequences and whole-genome assemblies. Int. J. Syst. Evol. Microbiol..

[50] Yang Y.J., Singh R.P., Lan X., Zhang C.S., Sheng D.H., Li Y.Q. (2019). Whole transcriptome analysis and gene deletion to understand the chloramphenicol resistance mechanism and develop a screening method for homologous recombination in Myxococcus xanthus. Microb. Cell Factories.

[51] Bolger A.M., Lohse M., Usadel B. (2014). Trimmomatic: A flexible trimmer for Illumina sequence data. Bioinformatics.

[52] Robinson M.D., McCarthy D.J., Smyth G.K. (2010). edgeR: A Bioconductor package for differential expression analysis of digital gene expression data. Bioinformatics.

[53] Mao X., Cai T., Olyarchuk J.G., Wei L. (2005). Automated genome annotation and pathway identification using the KEGG Orthology (KO) as a controlled vocabulary. Bioinformatics.

[54] Yang Y.J., Singh R.P., Lan X., Zhang C.S., Li Y.Z., Li Y.Q., Sheng D.H. (2018). Genome Editing in Model Strain Myxococcus xanthus DK1622 by a Site-Specific Cre/loxP Recombination System. Biomolecules.

[55] Guo H., Wu Y., Hong C., Chen H., Chen X., Zheng B., Jiang D., Qin W. (2017). Enhancing digestibility of Miscanthus using lignocellulolytic enzyme produced by Bacillus. Bioresour. Technol..

[56] Yuan Y., Zou P., Zhou J., Geng Y., Fan J., Clark J., Li Y., Zhang C. (2019). Microwave-assisted hydrothermal extraction of non-structural carbohydrates and hemicelluloses from tobacco biomass. Carbohydr. Polym..

[57] Goris J., Konstantinidis K.T., Klappenbach J.A., Coenye T., Vandamme P., Tiedje J.M. (2007). DNA-DNA hybridization values and their relationship to whole-genome sequence similarities. Int. J. Syst. Evol. Microbiol..

[58] Kanehisa M., Araki M., Goto S., Hattori M., Hirakawa M., Itoh M., Katayama T., Kawashima S., Okuda S., Tokimatsu T. (2008). KEGG for linking genomes to life and the environment. Nucleic Acids Res..

[59] Singh R.P., Manchanda G., Maurya I.K., Maheshwari N.K., Tiwari P.K., Rai A.R. (2019). Streptomyces from rotten wheat straw endowed the high plant growth potential traits and agro-active compounds. Bio. Agri. Biotechnol..

