# Supplementary material for: Genomic, Transcriptomic and Enzymatic Insight into Lignocellulolytic System of a Plant Pathogen Dickeya sp. WS52 to Digest Sweet Pepper and Tomato Stalk
Source: Biomolecules. 2019 Nov 20;9(12):753. doi: 10.3390/biom9120753 (PMC6995524; doi:10.3390/biom9120753)
Supplement: Supplementary file 1 [file biomolecules-09-00753-s001.pdf]

Title: Genomic, transcriptomic and enzymatic insight into lignocellulolytic system of a plant pathogen *Dickeya* sp. WS52 to digest sweet pepper and tomato stalk

Yingjie Yang[1,†], Wei Lin[2,†], Raghvendra Pratap Singh[3,*], Qian Xu[2], Zhihou Chen[2], Yuan Yuan[1], Ping Zou[1], Yiqiang Li[1] and Chengsheng Zhang[1,*]

1 Marine Agriculture Research Center, Tobacco Research Institute, Chinese Academy of Agricultural Sciences, Qingdao, Shandong 266101, China

YJY: yingjieyoung@sina.com; YY: yuanyuan03@caas.cn; PZ: zouping@caas.cn; CSZ: zhchengsheng@126.com; YL: liyiqiang@caas.cn.

2 Tobacco Research Institute of Nanping, Nangping, Fujian, 353000, China

WL: lw160718@163.com; QX:xq711126@163.com; ZHC: hhh0599@163.com

3 Department of Research & Development cell, Biotechnology, Uttaranchal University, Dehradun 248007, India RPS: rpsingh@uttaranchaluniversity.ac.in

*To whom correspondence should be addressed: rpsingh@uttaranchaluniversity.ac.in (R.P. Singh); zhangchengsheng@caas.cn (C. S. Zhang)

† These authors contributed equally to this work.

List of supporting materials

Figure S1. the surface symptom (Figure S1A) and the inner symptom (Figure S1B) of soft-rotten tobacco plant infected by Dickeya sp. WS52.

Figure S2. Classification of Raw Reads of three samples (A) Classification of Raw Reads of LB; (B) Classification of Raw Reads of Pep; (C) Classification of Raw Reads of Tmt.

Figure S3. Reads density in chromosome of three samples (A) Reads density of LB in chromosome; (B) Reads density of Pep in chromosome; (C) Reads density of Tmt in chromosome.

Figure S4. Distribution of reads mapped to genomic regions of three samples. (A) Distribution of reads mapped to genomic regions in LB; (B) Distribution of reads mapped to genomic regions in Pep; (C) Distribution of reads mapped to genomic regions in Tmt.

Figure S5. FPKM distribution for all samples

Figure S6. The correlation between samples. (A) the correlation in LB vs Pep; (B) the correlation in LB vs Tmt; (C) the correlation in Pep vs Tmt; (D) Pearson correlation between samples.

Figure S7. the overall distribution of DEGs in three samples. (A) LB vs Pep; (B) LB vs Tmt; (C) Pep vs Tmt.

Figure S8. Comparisons of the number and overlapping relationships of DEGs between different samples. (A) the total regulated DEGs shown in Ven map; (B) the upregulated DEGs shown in Ven map; (C) the downregulated DEGs shown in Ven map. Purple circle represents number of DEGs between Tmt vs Pep; yellow circle stand for number of DEGs between Pep vs LB; blue circle stand for number of DEGs between Tmt vs LB. The overlapping region means shared DEGs of two comparable groups.

Figure S9. The most enriched GO terms between Pep and Tmt.

Table S1 the identification of strain WS52 based on ANI

Table S2 transcriptome data output of three samples

Table S3 percentages of genes in different expression levels

Table S4 percentages of reads mapping to the reference genome

Table S5 the transcriptional level of potentially lignin degradation related genes in MSM + vegetable stalks compared to LB medium

Table S6 the transcriptome of ribosomal proteins in MSM + pepper or tomato stalk compared to LB medium

Table S7 the transcriptome of flagellar-forming proteins in MSM + pepper or tomato stalk compared to LB medium

Table S8 the transcriptome related with capsular in MSM + pepper or tomato stalk compared to LB medium

Table S9 the transcriptome of type II secretion system protein in MSM + pepper or tomato stalk compared to LB medium

Table S10 the transcriptome of type III secretion system protein in MSM + pepper or tomato stalk compared to LB medium

Table S11 the transcriptome of type VI secretion system protein in MSM + pepper or tomato stalk compared to LB medium

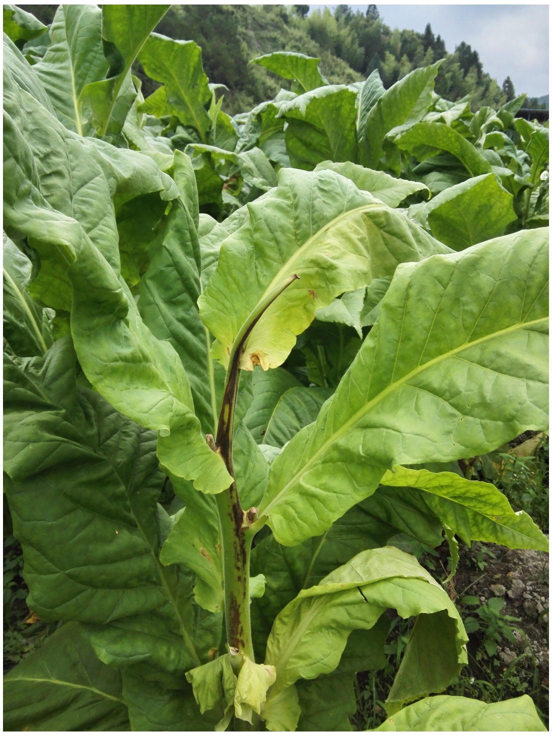

Figure S1A

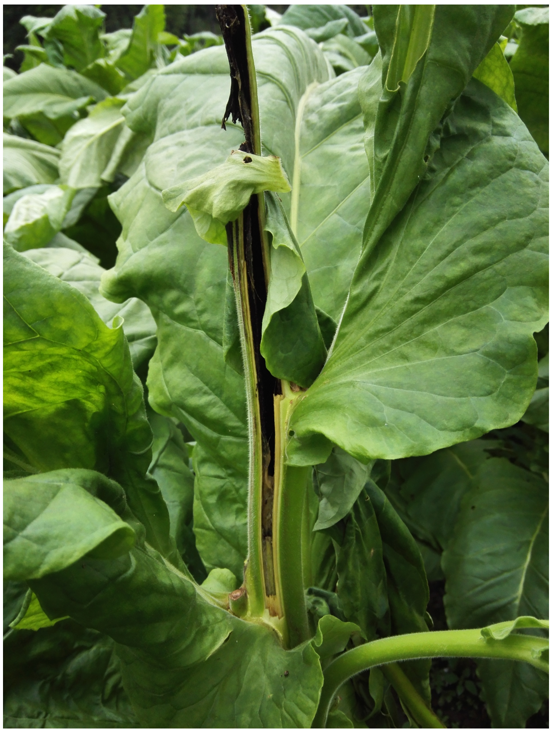

Figure S1B

Figure S1: the surface symptom (Figure S1A) and the inner symptom (Figure S1B) of soft-rotten tobacco plant infected by *Dickeya* sp. WS52.

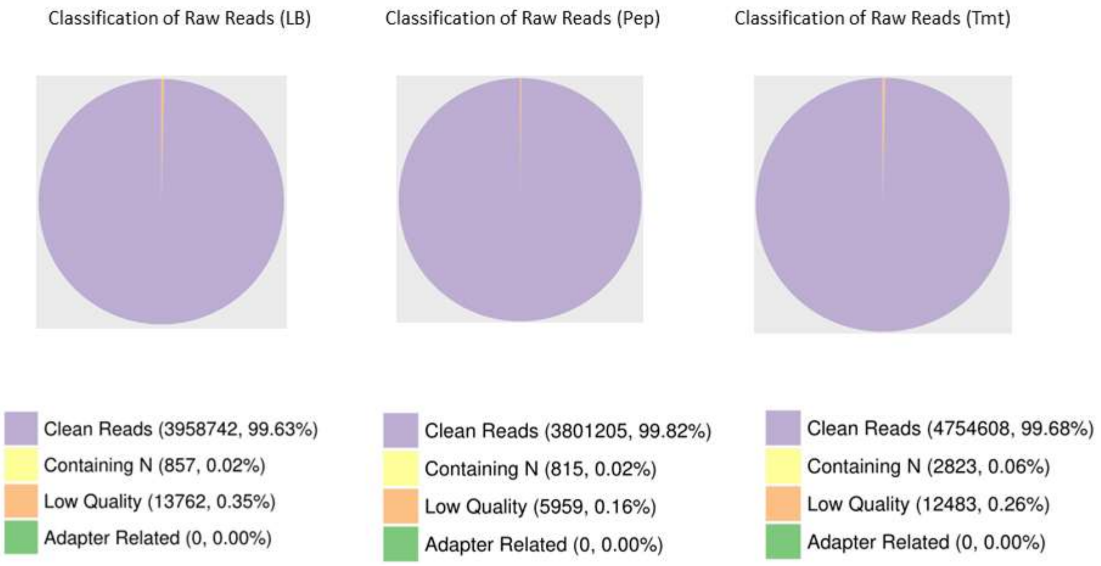

Figure S2. Classification of Raw Reads of three samples (A) Classification of Raw Reads of LB; (B) Classification of Raw Reads of Pep; (C) Classification of Raw Reads of Tmt.

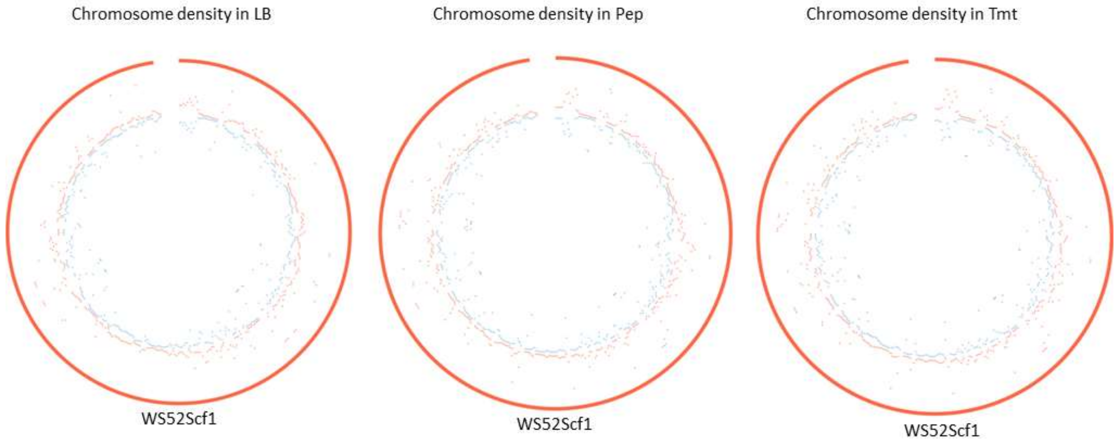

Figure S3. Reads density in chromosome of three samples (A) Reads density of LB in chromosome; (B) Reads density of Pep in chromosome; (C) Reads density of Tmt in chromosome.

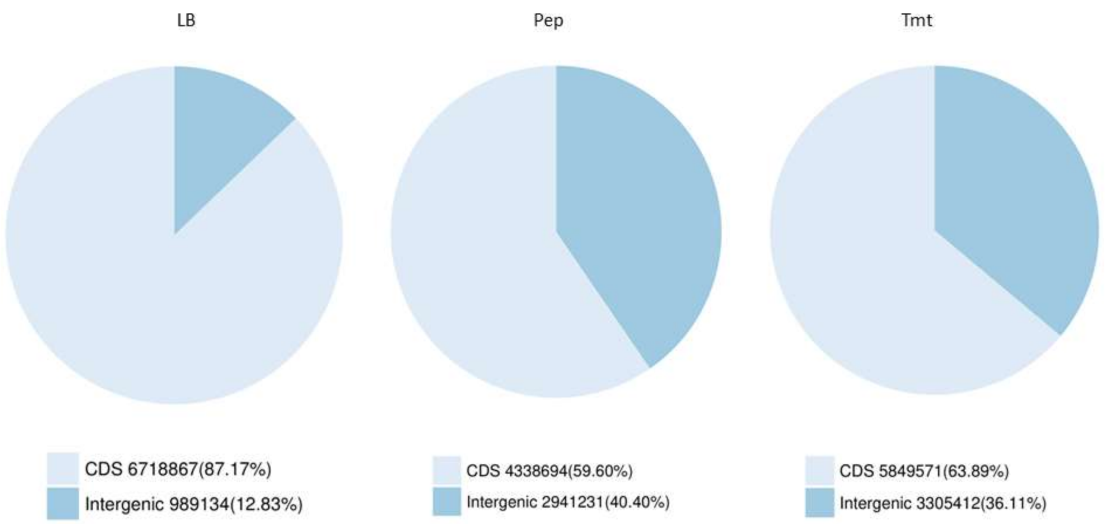

Figure S4. Distribution of reads mapped to genomic regions of three samples. (A) Distribution of reads mapped to genomic regions in LB; (B) Distribution of reads mapped to genomic regions in Pep; (C) Distribution of reads mapped to genomic regions in Tmt.

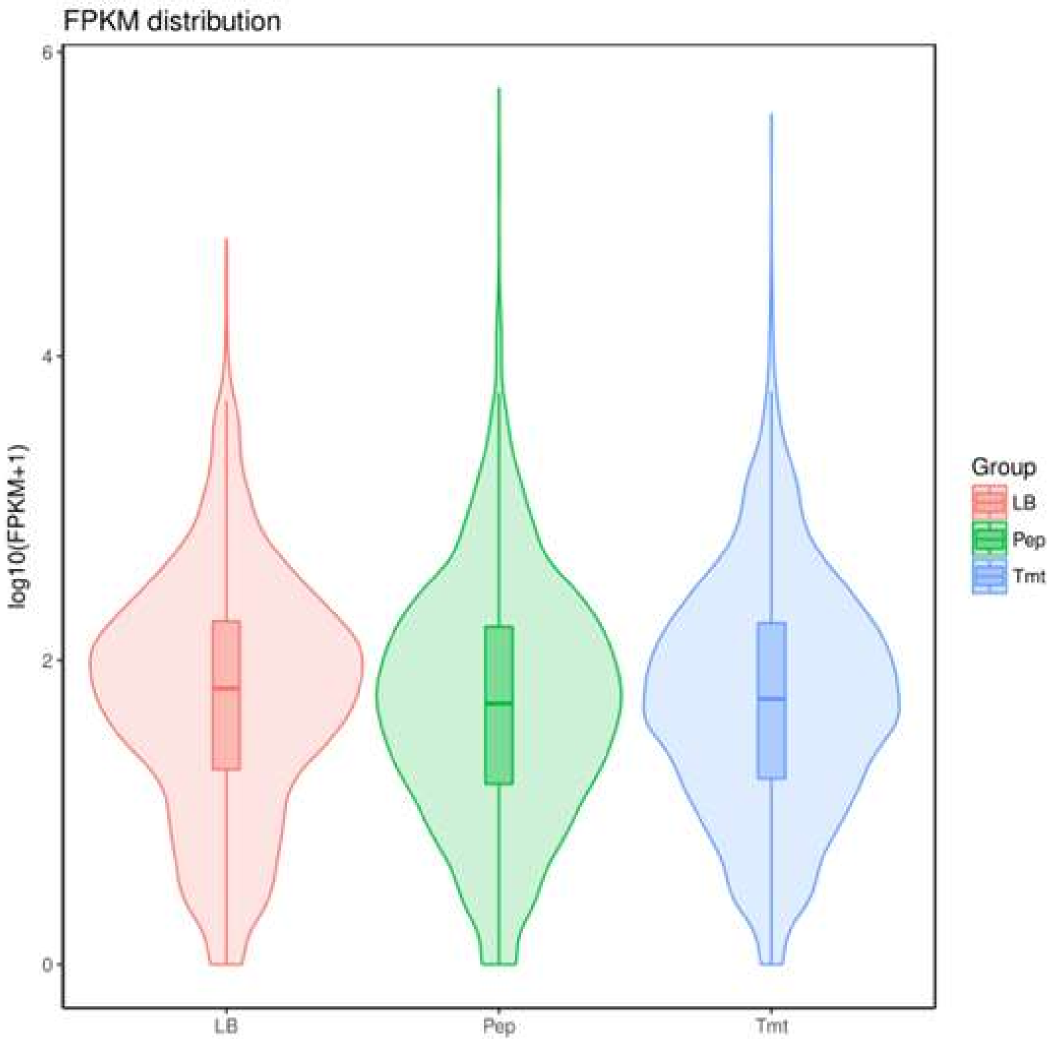

Figure S5. FPKM distribution for all samples

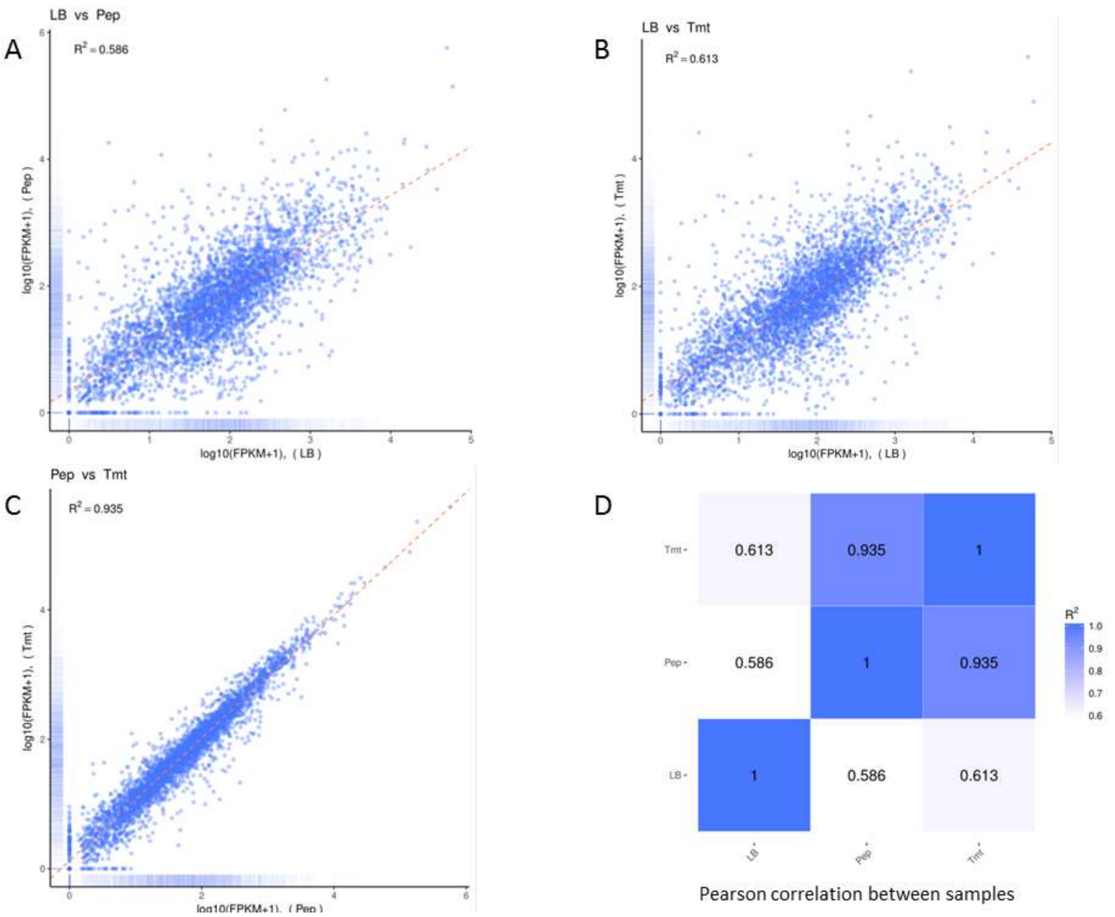

Figure S6. The correlation between samples. (A) the correlation in LB vs Pep; (B) the correlation in LB vs Tmt; (C) the correlation in Pep vs Tmt; (D) Pearson correlation between samples.

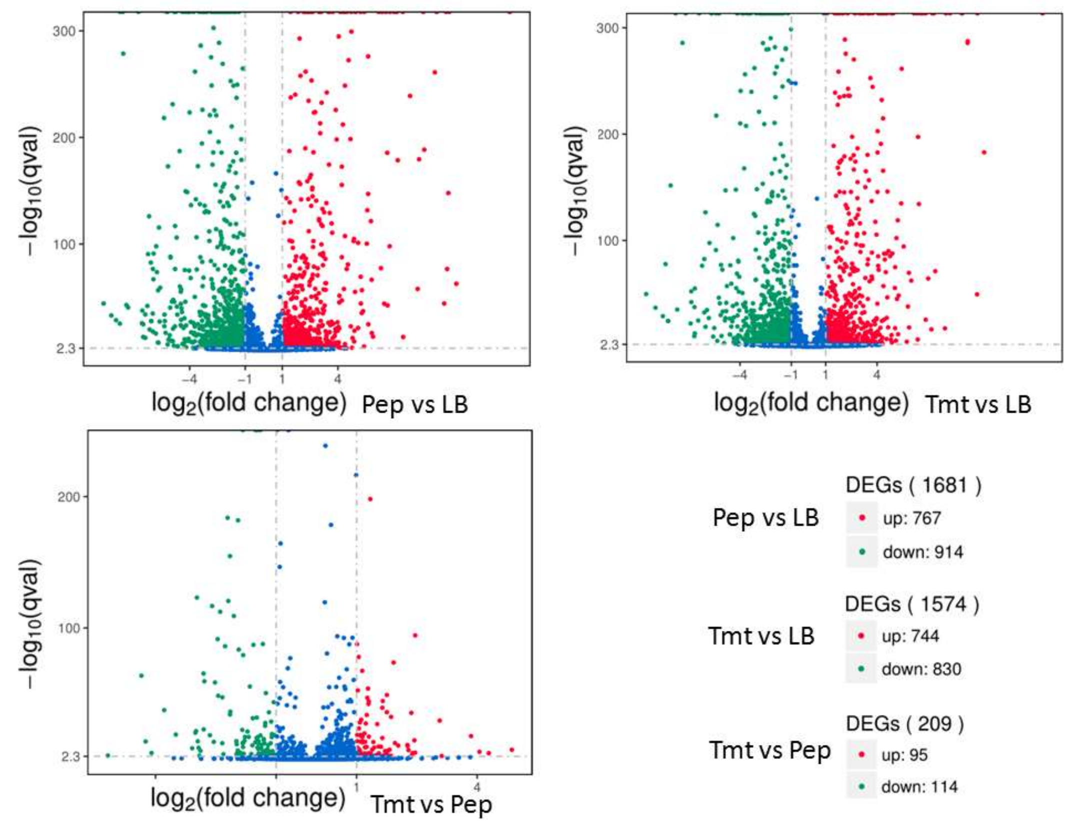

Figure S7. the overall distribution of DEGs in three samples. (A) LB vs Pep; (B) LB vs Tmt; (C) Pep vs Tmt.

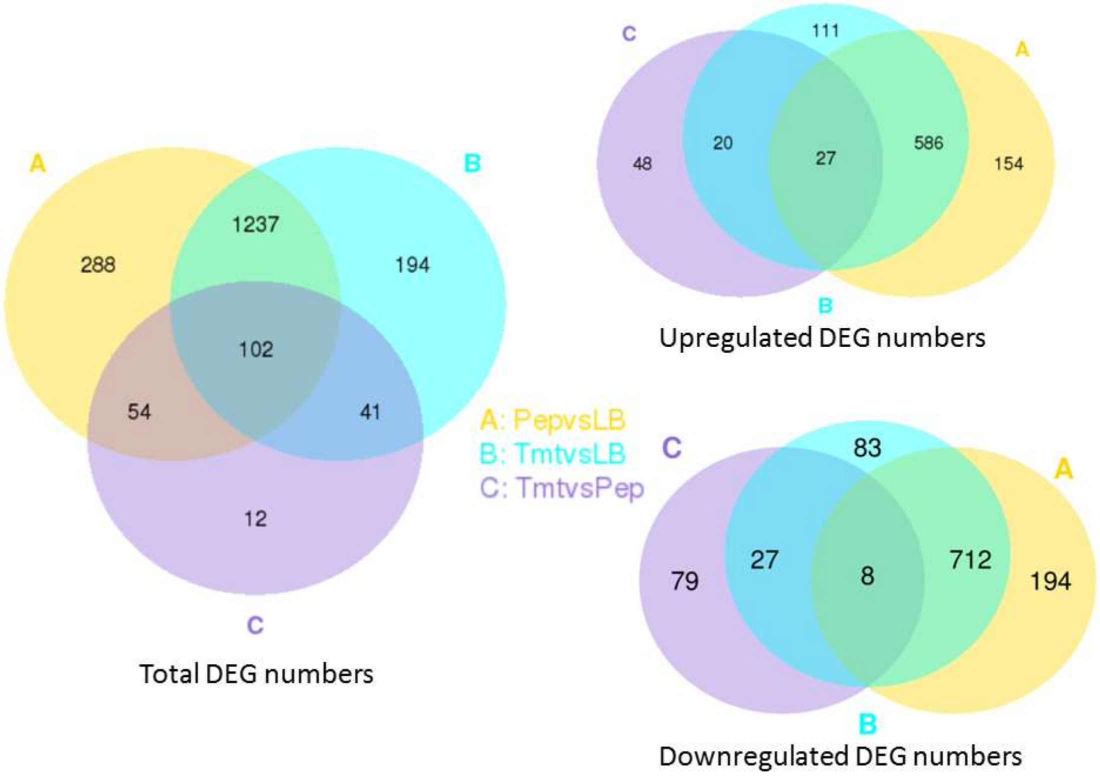

Figure S8. Comparisons of the number and overlapping relationships of DEGs between different samples. (A) the total regulated DEGs shown in Ven map; (B) the upregulated DEGs shown in Ven map; (C) the downregulated DEGs shown in Ven map. Purple circle represents number of DEGs between Tmt vs Pep; yellow circle stand for number of DEGs between Pep vs LB; blue circle stand for number of DEGs between Tmt vs LB. The overlapping region means shared DEGs of two comparable groups.

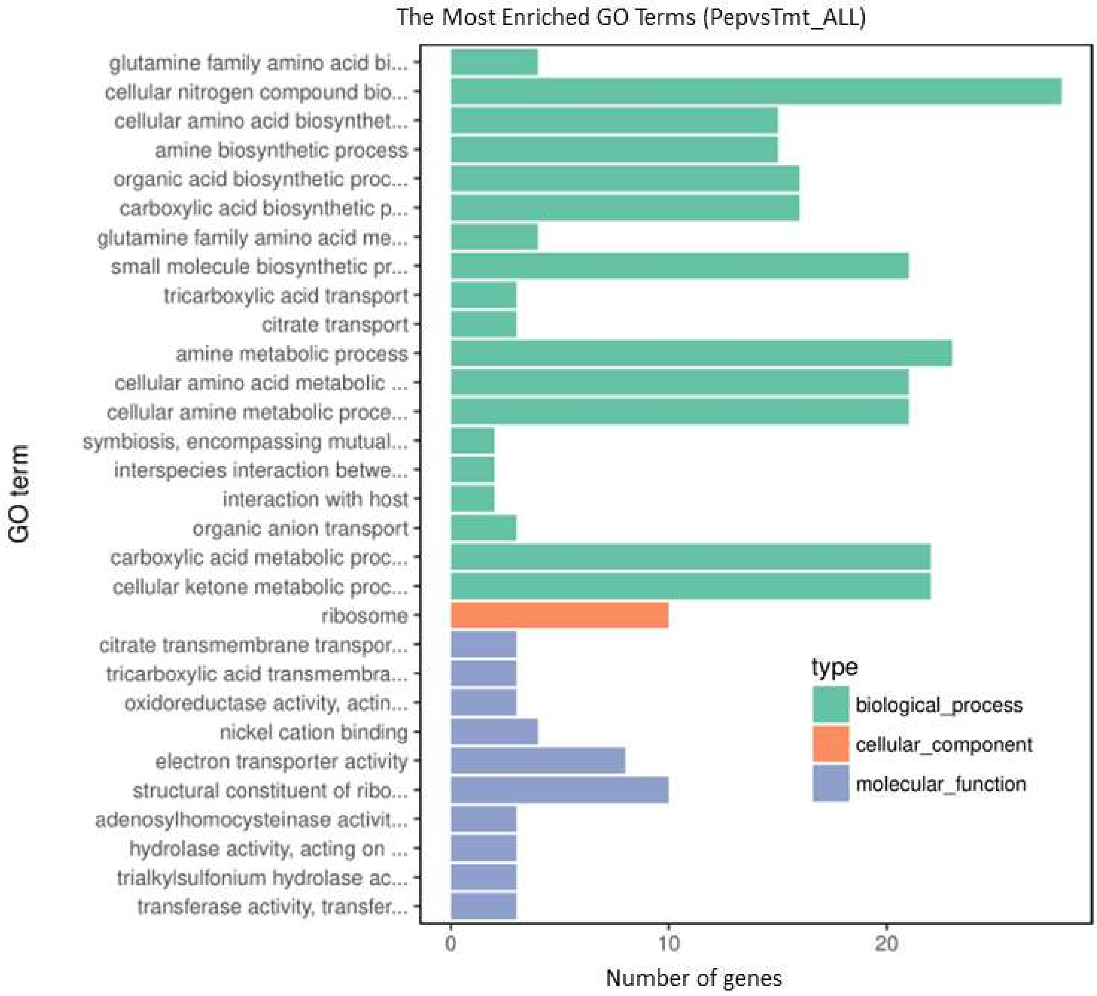

Figure S9 The most enriched GO terms between Pep and Tmt.

Table S1 the identification of strain WS52 based on ANI

strains	ANI
Dickeya_sp. 52	100
Dickeya_chrysanthemi_NCPPB_3533	98.4462
Dickeya_chrysanthemi_NCPPB_516	97.8787
Dickeya_chrysanthemi_NCPPB_402	96.4491
Dickeya_dadantii_NCPPB_898	86.716
Dickeya_dianthicola_NCPPB_453	86.6042
Dickeya_sp._NCPPB_3274	86.5572
Dickeya_solani_IPO_2222	86.451
Dickeya_zeae_Ech586	85.8197
Dickeya_zeae_NCPPB_3532	85.7944
Dickeya_zeae_NCPPB_2538	85.6397
Dickeya_sp._DW_0440	79.8501
Dickeya_sp_CSL_RW240	79.8201
Pectobacterium_atrosepticum_strain_NCPPB_549	75.2923
Pectobacterium_wasabiae RNS08.42.1A	75.2704
Pectobacterium_wasabiae_CFBP_3304	75.2322
Pectobacterium_carotovorum_brasiliense_LMG21371	75.5554

Table S2 transcriptome data output of three samples

Sample	Raw Reads	Raw Bases	Clean Reads	Clean Bases	Error Rate	Q20	Q30	GC Content
LB_1	3973361	0.59G	3958742	0.59G	0.01%	98.76%	96.15%	53.41%
LB_2	3973361	0.59G	3958742	0.59G	0.01%	97.57%	93.87%	52.90%
Pep_1	3807979	0.57G	3801205	0.57G	0.01%	98.79%	96.24%	52.63%
Pep_2	3807979	0.57G	3801205	0.57G	0.01%	98.22%	95.28%	52.21%
Tmt_1	4769914	0.71G	4754608	0.71G	0.01%	99.03%	96.76%	52.74%
Tmt_2	4769914	0.71G	4754608	0.71G	0.01%	98.39%	95.65%	52.33%

Table S3 percentages of reads mapping to the reference genome

Sample name	LB	Pep	Tmt
Total reads	7917484	7602410	9509216
Total mapped	7825522 (98.84%)	7494664 (98.58%)	9403391 (98.89%)
Multiple mapped	251101 (3.17%)	383899 (5.05%)	433671 (4.56%)
Uniquely mapped	7574421 (95.67%)	7110765 (93.53%)	8969720 (94.33%)
Read-1	3785604 (47.81%)	3556456 (46.78%)	4484768 (47.16%)
Read-2	3788817 (47.85%)	3554309 (46.75%)	4484952 (47.16%)
Reads map to '+'	3787545 (47.84%)	3554772 (46.76%)	4484603 (47.16%)
Reads map to '-'	3786876 (47.83%)	3555993 (46.77%)	4485117 (47.17%)

Table S4 percentages of genes in different expression levels

FPKM Interval	LB	Pep	Tmt
0~1	191(4.60%)	192(4.62%)	141(3.40%)
1~3	228(5.49%)	205(4.94%)	216(5.20%)
3~15	579(13.95%)	726(17.49%)	687(16.55%)
15~60	1043(25.12%)	1121(27.00%)	1138(27.41%)
>60	2111(50.84%)	1908(45.95%)	1970(47.45%)

Table S5 the transcriptional level of potentially lignin-degradation related genes in MSM + vegetable stalks compared to LB medium

ORF number (TYL)	log2FC in Pep	log2FC in Tmt	annotation
44660.1	3.19	2.10	(AA2)multicopper oxidase
44482.1	3.33	3.69	(AA3)GMC family oxidoreductase
43757.1	n.d.	n.d.	(AA6)flavodoxin family protein
41583.1	n.d.	n.d.	(AA6)flavodoxin family protein
43996.1	n.d.	n.d.	hydrogen peroxide-inducible genes activator
43983.1	n.d.	n.d.	quinone oxidoreductase
43984.1	-1.25	n.d.	quinone oxidoreductase
43985.1	n.d.	n.d.	quinone oxidoreductase
43986.1	n.d.	n.d.	quinone oxidoreductase
43987.1	n.d.	n.d.	quinone oxidoreductase
43988.1	n.d.	n.d.	quinone oxidoreductase
43989.1	n.d.	n.d.	quinone oxidoreductase
43990.1	-1.10	-1.20	quinone oxidoreductase
43991.1	-1.22	-1.30	quinone oxidoreductase
43992.1	-1.01	n.d.	quinone oxidoreductase
43993.1	-1.31	n.d.	quinone oxidoreductase
43994.1	n.d.	n.d.	quinone oxidoreductase
43995.1	n.d.	n.d.	quinone oxidoreductase

44756.1	n.d.	n.d.	glutathione S-transferases
44033.1	1.49	n.d.	glutathione S-transferases
43409.1	n.d.	n.d.	glutathione S-transferases
42345.1	1.84;	2.26	glutathione S-transferases
41649.1	n.d.	n.d.	glutathione S-transferases
41540.1	-2.12;	-2.16	glutathione S-transferases
41013.1	n.d.	n.d.	glutathione S-transferases
41021.1	n.d.	n.d.	glutathione S-transferases
43777.1	n.d.	n.d.	catalase-peroxidase
43797.1	-2.60	-1.89	Peroxidase
43245.1	2.98	4.76	glutathione peroxidase
43246.1	n.d.	n.d.	hydrogen peroxide-inducible genes activator
43612.1	n.d.	n.d.	glutathione peroxidase
41016.1	n.d.	n.d.	glutathione peroxidase

Table S6 the transcriptome of ribosomal proteins in MSM + pepper or tomato stalk compared to LB medium

No. of orf (TYL)	log2FC in Pep	log2FC in Tmt	Annotation
40843.1	-2.56	-1.48	50S ribosomal protein L17
40844.1	-2.61	-1.66	DNA-directed RNA polymerase subunit alpha
40845.1	-2.21	-1.39	30S ribosomal protein S4
40846.1	-1.83	-1.32	30S ribosomal protein S11, partial
40847.1	-1.72	-1.39	30S ribosomal protein S13
40848.1	-1.11	-1.02	50S ribosomal protein L36
40849.1	-1.83	-1.46	preprotein translocase subunit SecY
40850.1	-2.84	-1.97	50S ribosomal protein L15
40852.1	-3.23	-2.34	30S ribosomal protein S5
40853.1	-3.19	-2.17	50S ribosomal protein L18
40854.1	-3.21	-1.93	50S ribosomal protein L6
40855.1	-2.76	-1.72	30S ribosomal protein S8
40856.1	-2.12	-1.19	30S ribosomal protein S14
40857.1	-1.73	-1.05	50S ribosomal protein L5
40858.1	-1.25	-1.17	50S ribosomal protein L24
40859.1	-1.63	-1.79	50S ribosomal protein L14
40860.1	-2.34	-1.65	30S ribosomal protein S17
40861.1	-2.19	-2.39	50S ribosomal protein L29
40862.1	-3.34	-2.78	50S ribosomal protein L16
40863.1	-4.00	-2.50	30S ribosomal protein S3
40864.1	-4.13	-2.50	50S ribosomal protein L22
40865.1	-4.79	-2.59	30S ribosomal protein S19
40866.1	-4.26	-1.58	50S ribosomal protein L2
40867.1	-2.85	-1.98	50S ribosomal protein L23
40868.1	-3.23	-1.94	50S ribosomal protein L4

40869.1	-2.82	-1.85	50S ribosomal protein L3
40870.1	-2.56	-1.97	30S ribosomal protein S10

Table S7 the transcriptome of flagellar-forming proteins in MSM + pepper or tomato stalk compared to LB medium

No. of orf (TYL)	log2FC in Pep	log2FC in Tmt	Annotation
43505.1	2.26	2.07	flagellar transcriptional activator FlhD
43506.1	1.57	1.55	flagellar transcriptional activator FlhC
43507.1	2.05	2.24	flagellar motor protein MotA
43507.1	1.15	1.69	flagellar motor protein MotB
43508.1	1.46	1.12	chemotaxis protein CheW
43510.1	1.90	1.81	Hemotaxis protein-glutamate O-methyltransferase
43514.1	2.37	2.56	flagellar biosynthesis protein FlhB
43517.1	2.63	2.65	flagellar biosynthesis protein Flg
43518.1	3.54	3.21	flagellar biosynthesis protein FlgM
43539.1	1.72	1.11	flagellar protein FliJ
43545.1	2.65	3.34	flagellar protein FliS
43546.1	2.98	3.02	flagellar hook protein FliD

Table S8 the transcriptome related with capsular in MSM + pepper or tomato stalk compared to LB medium

No. of orf (TYL)	log2FC in Pep	log2FC in Tmt	Annotation
42892.1	-3.04	-2.74	capsular biosynthesis protein
42893.1	-3.10	-2.64	capsular biosynthesis protein
42894.1	-2.55	-2.32	capsular biosynthesis protein
42895.1	-1.66	-3.55	capsular biosynthesis protein
42896.1	-3.29	-1.25	capsular polysaccharide biosynthesis protein

Table S9 the transcriptome of type II secretion system protein in MSM + pepper or tomato stalk compared to LB medium

No. of orf (TYL)	log2FC in Pep	log2FC in Tmt	Annotation
44086.1	-1.60	-1.72	type II secretion protein M
44087.1	-1.66	-2.08	type II secretion system protein GspL
44088.1	-1.32	-2.65	Type II secretion system protein K; Pectic enzymes secretion protein OutK
44089.1	-1.64	-2.82	general secretion pathway protein J
44090.1	-1.69	-2.60	type II secretion system protein GspI
44091.1	-2.20	-2.94	general secretion pathway protein H
44092.1	-3.12	-4.20	type II secretion system protein GspG
44093.1	-2.13	-3.40	type II secretion system protein GspF

44094.1	-1.90	-2.51	type II secretion system protein GspE
44095.1	-1.41	-2.13	type II secretion system protein GspD
44096.1	-2.04	-1.80	type II secretion system protein GspC
41260.1	2.79	3.67	type II secretion system protein GspD
41262.1	2.25	2.82	general secretion pathway protein GspF
41266.1	2.02	2.06	general secretion pathway protein GspL

Table S10 the transcriptome of type III secretion system protein in MSM + pepper or tomato stalk compared to LB medium

No. of orf (TYL)	log2FC in Pep	log2FC in Tmt	Annotation
41618.1	2.43	2.51	EscC/YscC/HrcC family type III secretion system outer membrane ring protein
41701.1	2.64	3.08	type III secretion apparatus lipoprotein, YscJ/HrcJ family
44125.1	-2.55	-2.59	type III secretion system effector
44126.1	-1.85	-1.63	virulence factor SrfB

Table S11 the transcriptome of type VI secretion system protein in MSM + pepper or tomato stalk compared to LB medium

No. of orf (TYL)	log2FC in Pep	log2FC in Tmt	Annotation
43807.1	-5.95	-5.40	type VI secretion protein IcmF
43808.1	-6.50	-6.44	type VI secretion-associated protein
43809.1	-5.14	-6.67	type VI secretion-associated protein
43810.1	-7.41	-8.93	T6SS ATPase
43811.1	-5.37	-6.89	chemotaxis protein MotB
43812.1	-7.32	-6.84	type VI secretion protein
43813.1	-6.50	-6.64	type VI secretion lipoprotein
43814.1	-7.76	-8.20	type VI secretion protein
43815.1	-8.23	-7.67	type VI secretion protein
43816.1	-5.98	-6.22	type VI secretion protein
43817.1	-6.56	-6.66	type VI secretion protein
43879.1	-7.60	-7.76	type VI secretion protein
43818.1	-5.20	-5.66	type VI secretion protein
42936.1	-2.59	-4.01	type VI secretion system protein
43831.1	-2.32	-2.14	type VI secretion system tip protein VgrG
41282.1	-6.39	-8.50	type VI secretion protein VgrG
41118.1	-3.72	-5.57	type VI secretion protein VgrG
